# Aberrant paracrine signalling for bone remodelling underlies the mutant histone-driven giant cell tumour of bone

**DOI:** 10.1038/s41418-022-01031-x

**Published:** 2022-08-03

**Authors:** Lucia Cottone, Lorena Ligammari, Hang-Mao Lee, Helen J. Knowles, Stephen Henderson, Sara Bianco, Christopher Davies, Sandra Strauss, Fernanda Amary, Ana Paula Leite, Roberto Tirabosco, Kristian Haendler, Joachim L. Schultze, Javier Herrero, Paul O’Donnell, Agamemnon E. Grigoriadis, Paolo Salomoni, Adrienne M. Flanagan

**Affiliations:** 1 Department of Pathology, UCL Cancer Institute, University College London, London, WC1E 6BT UK; 2 German Center for Neurodegenerative Diseases (DZNE), 53127 Bonn, Germany; 3 Botnar Institute for Musculoskeletal Sciences, Nuffield Department of Orthopaedics Rheumatology & Musculoskeletal Sciences, University of Oxford, Oxford, OX3 7LD UK; 4 Bill Lyons Informatics Centre (BLIC), UCL Cancer Institute, University College London, London, WC1E 6BT UK; 5 Samantha Dickson Brain Cancer Unit, Department of Cancer Biology, UCL Cancer Institute, University College London, London, WC1E 6BT UK; 6 Department of Histopathology, Royal National Orthopaedic Hospital, Middlesex, Stanmore, HA7 4LP UK; 7 London Sarcoma Service, University College London Hospitals Foundation Trust, London, WC1E 6DD UK; 8 Platform for Single Cell Genomics and Epigenomics (PRECISE) at the DZNE and the University of Bonn, 53127 Bonn, Germany; 9 Institute of Human Genetics, University of Lübeck, Lübeck, Germany; 10 Genomics and Immunoregulation, Life and Medical Sciences (LIMES) Institute, University of Bonn, 53115 Bonn, Germany; 11 Department of Radiology, Royal National Orthopaedic Hospital, Middlesex, Stanmore, HA7 4LP UK; 12 Centre for Craniofacial and Regenerative Biology, King’s College London, Guy’s Hospital, London, SE1 9RT UK

**Keywords:** Epigenetics, Sarcoma, Cancer microenvironment

## Abstract

Oncohistones represent compelling evidence for a causative role of epigenetic perturbations in cancer. Giant cell tumours of bone (GCTs) are characterised by a mutated histone H3.3 as the sole genetic driver present in bone-forming osteoprogenitor cells but absent from abnormally large bone-resorbing osteoclasts which represent the hallmark of these neoplasms. While these striking features imply a pathogenic interaction between mesenchymal and myelomonocytic lineages during GCT development, the underlying mechanisms remain unknown. We show that the changes in the transcriptome and epigenome in the mesenchymal cells caused by the H3.3-G34W mutation contribute to increase osteoclast recruitment in part via reduced expression of the TGFβ-like soluble factor, SCUBE3. Transcriptional changes in *SCUBE3* are associated with altered histone marks and H3.3^G34W^ enrichment at its enhancer regions. In turn, osteoclasts secrete unregulated amounts of SEMA4D which enhances proliferation of mutated osteoprogenitors arresting their maturation. These findings provide a mechanism by which GCTs undergo differentiation in response to denosumab, a drug that depletes the tumour of osteoclasts. In contrast, *hTERT* alterations, commonly found in malignant GCT, result in the histone-mutated neoplastic cells being independent of osteoclasts for their proliferation, predicting unresponsiveness to denosumab. We provide a mechanism for the initiation of GCT, the basis of which is dysfunctional cross-talk between bone-forming and bone-resorbing cells. The findings highlight the role of tumour/microenvironment bidirectional interactions in tumorigenesis and how this is exploited in the treatment of GCT.

## Introduction

Giant cell tumour of bone (GCT) is a locally aggressive primary neoplasm of bone [1]. At the genetic level it is characterised by the presence of a near universal H3.3 Histone A (*H3-3A*) G34W missense mutation (H3.3^G34W^) which represents the sole genetic driver [2]. Transformation of conventional to malignant GCT requires acquisition of at least one additional driver alteration, commonly in Telomerase Reverse Transcriptase (*hTER*T), reflected in histological characteristics of high grade sarcoma [3].

GCT is composed of osteoprogenitors/stromal bone-forming cells of mesenchymal origin, which harbour the H3.3^G34W^ mutations and constitute the neoplastic component, and a pronounced tumour microenvironment (TME) dominated by unmutated osteoclasts [2]. Osteoclasts, multinucleated bone-resorbing cells formed by fusion of myeloid precursors (monocytes), in GCTs are unusually large containing up to 100 nuclei [1]. The mechanisms that lead to such a conspicuous osteoclast population are complex and have not yet been fully elucidated [4, 5], although there are reports implicating Receptor Activator of Nuclear Factor kappa-Β Ligand (RANKL) [6, 7] a molecule on which osteoclasts depend for their formation [8]. Denosumab, a humanised antibody to RANKL, which blocks osteoclast formation, is widely employed to reduce bone resorption in osteoporosis and localised osteolysis associated with metastatic cancer and is also used to control growth of GCT by targeting the TME [6].

Histone H3.3 is a replication-independent variant histone which facilitates transcription at euchromatic regions [9]. In addition to GCT, mutations in the histone H3.3 have been identified in gliomas (H3.3K27M or H3.3G34R/V) [10] and in chondroblastomas (H3.3K36M), a rare benign bone tumour [2]. Recent evidence suggests that H3.3^G34W^ promotes PRC2/H3K27me3 silencing of H3K36me3-depleted nucleosomes, H3.3 redistribution [11] and changes in the DNA methylation profile in osteoprogenitors [7]. This epigenetic remodelling is reported to alter mesenchymal lineage commitment, including stalling of the osteogenic differentiation process [7, 11, 12]. However, these studies do not explain fully how mutated osteoblasts influence osteoclastogenesis, a key pathogenic component of oncohistone-driven bone neoplasms. Here, we provide insights into how the H3.3^G34W^ mutation perturbs the finely tuned physiological interaction between bone-forming and bone-resorbing cells, implicating alteration of the epigenome of osteoprogenitors and the expression of a previously unknown modulator of osteoclastogenesis, *SCUBE3*.

## Results

### H3.3^G34W^ in osteoprogenitors regulates bone formation without affecting proliferation

**Fig. 1:**
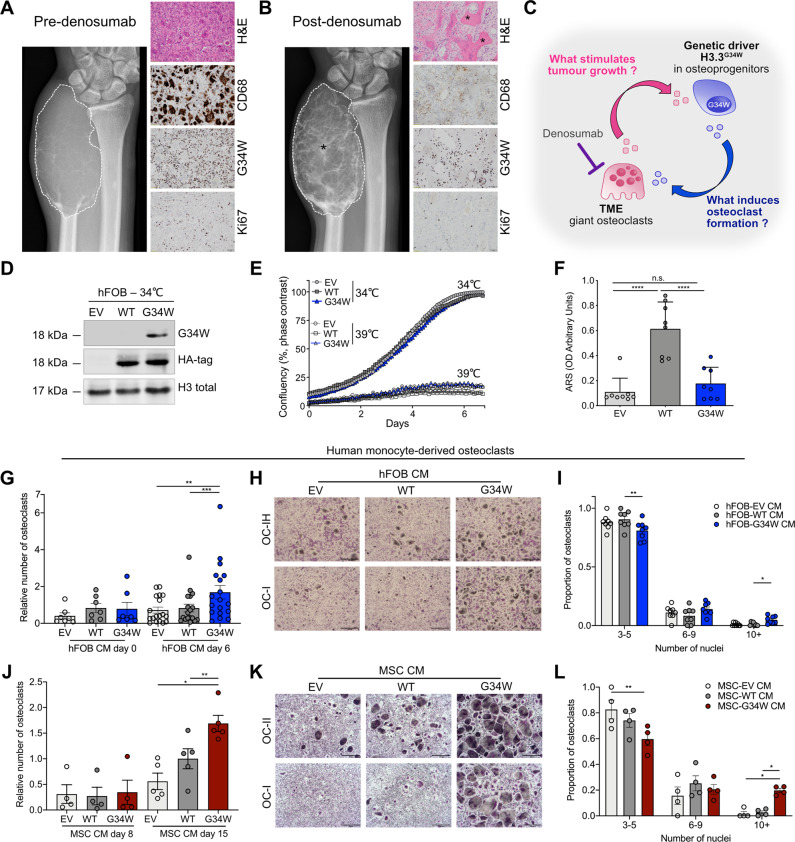
Mutant H3.3^G34W^ in osteoprogenitors regulates bone formation and stimulates osteoclast recruitment. **A**, **B** GCT of the distal ulna in a 35-year-old male. **A** Pre-denosumab treatment: anteroposterior radiograph of the distal ulna shows the tumour (dotted outline) without mineralisation. H&E-stained section: features of a benign GCT with CD68-positive-osteoclasts interspersed with proliferative H3.3^G34W^-mononuclear stromal cells (G34W; Ki67). **B** Post-denosumab treatment: after one month’s treatment showing similar tumour size (dotted outline) to A but prominent mineralisation (asterisk). H&E-section: bone formation (asterisk) and absence of CD68-positive osteoclasts but persistent H3.3^G34W^-neoplastic cells (G34W) with a reduced proliferative index (Ki67) compared to **A**. **C** Schema of proposed interactions between stromal/osteoprogenitors and TME. H3.3^G34W^-osteoprogenitor-derived factor(s) result in an environment permissive for formation of abnormally large osteoclasts, which secrete factor(s) stimulating tumour growth. Denosumab treatment results in depletion of osteoclasts and removes the growth stimulus for stromal/osteoprogenitors. **D** Representative western blot of HA-tagged-H3.3 and H3.3-G34W expression in transfected hFOBs. **E** Proliferation of undifferentiated hFOBs grown at 34 °C and differentiated at 39 °C for 6 days in mineralisation medium assessed by Incucyte; 2 experiments, 3 replicates per experiment. **F** Quantification of ARS mineralisation of hFOBs on day 6 of differentiation. 8 replicates, three experiments. **G**, **J** Number of osteoclasts generated in the presence of conditioned medium (CM) from (**G**) undifferentiated (day 0, 34  °C) and differentiated (at 39 °C for 6 days) hFOB (number relative to WT CM day 6) and (**J**) from iPSC-derived-MSCs differentiated to osteoblasts for 8 and 15 days (number relative to WT CM day 15). 7–18 (**G**) and 4-5 (**J**) osteoclast preparations. **H**, **K** Representative tartrate-resistant acid phosphatase (TRAP) staining of two osteoclast cultures (OC-I and II) in the presence of CM from (**H**) hFOBs differentiated for 6 days or (**K**) iPSC-derived-MSCs differentiated to osteoblasts for 15 days; 8 osteoclast preparations. 4× magnification. **I**, **L** Quantification of number of nuclei per osteoclast in **H** and **K** expressed as proportion of OCs, calculated as the number of OCs exhibiting 3–5, 6–9 or ≥10 nuclei over the total number of OCs; results from 8 (**I**) and 4 (**L**) osteoclast preparations. Data are mean ± SD (**F**), ±SEM (**G**, **J**, **I**, **L**). **F** 1-way ANOVA. **G**, **J**: 1-way repeated measures (RM)ANOVA for each time point. **I**, **L**: 2-way RM ANOVA.

To understand how H3.3^G34W^ drives the pathogenesis of GCT, we looked at the clinical impact of denosumab on these tumours: treatment not only results in depletion of osteoclasts but also causes a reduction in proliferation and an increase in differentiation/bone formation of the mutant osteoprogenitors [13] (Fig. 1A, B). This led us to consider that the GCT is driven by the H3.3^G34W^-mutant osteoprogenitors in part in a non-cell autonomous manner by recruiting large osteoclasts to the TME and co-opting them to secrete factors which provide the growth advantage to the tumour cells (Fig. 1C). To test this hypothesis, we stably expressed H3.3^G34W^, H3.3^WT^ and empty vector (EV) in vitro in an immortalised human fetal osteoblastic cell line (hFOB) (Fig. 1D and Supplementary Fig. 1A, B). hFOBs are temperature-sensitive osteoprogenitors which proliferate when cultured at 34 °C but differentiate in mature bone-forming osteoblasts at 39 °C.

Overexpression of H3.3^G34W^ did not alter hFOBs proliferation, migration or survival compared to H3.3^WT^ or EV, even after long-term passaging (Fig. 1E and Supplementary Fig. 1C–F). Instead, differentiation assays showed that while H3.3^WT^ enhanced bone formation, H3.3^G34W^ impaired this function (Fig. 1F and Supplementary Fig. 1G–H). Overexpression of H3.3^G34W^ also had no effect on proliferation or survival of osteoblasts differentiated from mesenchymal stem cells (MSCs) derived from induced from Pluripotent Stem Cells (iPSCs), another model of osteoblast differentiation (Supplementary Fig. 2A–M). Together, these results suggest that H3.3^G34W^ does not directly control growth of the stromal/osteoprogenitor cells, but unlike H3.3^WT^, impairs their differentiation.

### H3.3^G34W^ stimulates osteoclast recruitment

Using an in vitro osteoclastogenesis assay of human monocyte-derived osteoclasts, we next tested if the H3.3^G34W^ mutation in osteoprogenitors accounts for the prominent osteoclast population in GCT. We showed that the conditioned medium (CM) from differentiated (bone-forming) H3.3^G34W^-hFOBs significantly increased the number of osteoclasts and particularly those with more than 10 nuclei, albeit with variable results due to donor-to-donor variability (Fig. 1G–I). CM from differentiated H3.3^G34W^-stromal cells from iPSC-MSCs also induced a significant increase in large osteoclasts (Fig. 1J–L). This supports the concept that the driver mutation in stromal/osteoprogenitor cells is responsible for the osteoclast-rich phenotype of the GCT.

### H3.3^G34W^ affects transcription of osteoprogenitors and leads to downregulation of *SCUBE3*

**Fig. 2:**
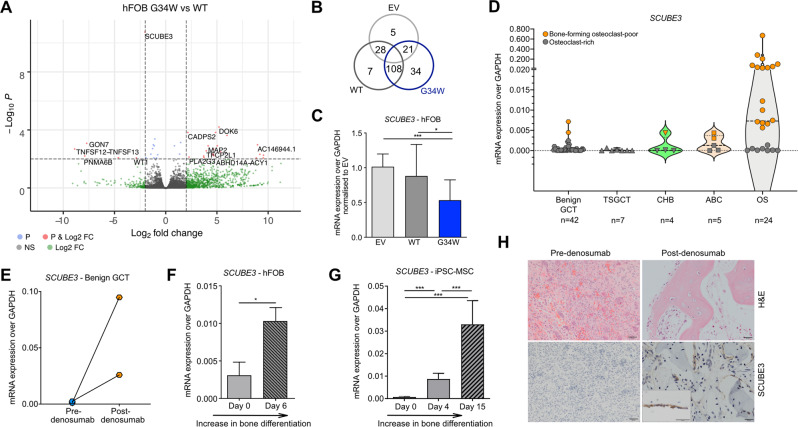
H3.3^G34W^ hFOB cells and GCTs have low levels of *SCUBE3*. **A** Volcano plot showing the relationship between the mean difference in gene expression by RNA-seq; *p* < 0.001 (i.e., −log10P > 3) and log fold Change >2. Total 35,639 variables. *P* is the Independent Hypothesis Weighting (IHW)-adjusted *p* value. **B** Venn diagram showing the number of differentially expressed genes, in hFOB transfectants, by RNA-seq. **C** Expression of *SCUBE3* by qPCR in hFOBs. 6 experiments from 4 independent infections, 2 replicates each experiment. **D** Expression of *SCUBE3* by qPCR in GCT and GCT-mimics: benign GCT, TSGCT-tenosynovial giant cell tumour, CHB-chondroblastoma, ABC-aneurysmal bone cyst and OS-osteosarcoma. Grey: osteoclast-rich samples. Orange: bone-forming osteoclast-poor samples. Dashed lines: median and quartiles. **E** Expression of *SCUBE3* by qPCR in benign GCT samples pre- and post-denosumab treatment. Data from 2 patients: post-treatment (orange) and their corresponding osteoclast-rich pre-treatment samples (light blue). **F**, **G** Expression of *SCUBE3* by qPCR in (**F**) H3.3^WT^ hFOB undifferentiated and after 6 days of differentiation (6 replicates, 2 experiments) and in (**G**) H3.3^WT^ iPSC-derived-MSCs during osteoblast maturation (6 replicates, 2 experiments). **H** H&E-stained sections showing SCUBE3 expression in mature bone-lining cells of post-denosumab-treatedGCT patients’ samples; 20× magnification. Data are mean ± SD. **C**, **F**: 1-way ANOVA. **E**: unpaired Student’s *t* test.

We next sought the molecule(s) underlying the osteoclast-inducing effect by performing bulk RNA-sequencing (RNA-seq) of H3.3^G34W^ hFOB and control lines (Fig. 2A, B, Supplementary Fig. 3A–C and Supplementary Data 1).

We found that H3.3^G34W^ had no impact on the expression of *RANKL* and *Osteoprotegerin*, as previously described [12], and other critical cytokines reported to be involved in osteoclast formation including *TNFSF13* [14] (Supplementary Fig. 3C, D and Supplementary Data 1). Instead, the *Signal Peptide CUB Domain And EGF Like Domain Containing 3 (SCUBE3)*, a secreted member of the transforming growth factor beta (TGFβ) family, was the most significantly downregulated gene in H3.3^G34W^ compared to H3.3^WT^ cells (Fig. 2A, C). SCUBE3, which acts as an endogenous ligand for TGFβ-receptor 2 [15], is a valid candidate for regulating osteoclast recruitment: it is expressed by osteoblasts [16] (Supplementary Fig. 3E), its inactivation results in a syndrome characterised by impaired ossification in humans and mouse models [17, 18], and it is linked to Paget’s disease of bone which is characterised by numerous large osteoclasts [16]. Consistent with our in vitro results, *SCUBE3* mRNA was virtually absent in GCT samples and in other osteoclast-rich tumours (Fig. 2D). In contrast, *SCUBE3* mRNA levels were increased in post-denosumab-treated GCTs compared to untreated tumours and were also high in bone-forming osteoclast-poor osteosarcomas compared to osteoclast-rich osteosarcomas (Fig. 2D, E). These data indicate an inverse correlation between osteoprogenitor *SCUBE3* expression and osteoclast activity across several bone tumour types, an effect potentially related to the state of maturation of bone cells. This is supported by higher levels of *SCUBE3* in osteoblasts undergoing differentiation in vitro (Fig. 2F, G) and by the localisation of SCUBE3 expression to bone-forming cells in human samples of mature bone (Fig. 2H).

### H3.3^G34W^ alters histone marks at *SCUBE3* and genome-wide in osteoprogenitors

We next investigated whether H3.3^G34W^ affected alterations in histone marks at the *SCUBE3* locus and at the genome-wide level by profiling HA-tagged H3.3 in hFOB cells by ChIP-sequencing normalised with an exogenous reference genome (ChIP-Rx) (Supplementary Fig. 4A, B and Supplementary Data 2 and 3). We also profiled the H3K27ac and the H3K36me3 marks which previously have been found altered in H3.3^G34R^-mutant brain tumours [19, 20] and in H3.3^G34W^ GCT-derived cell lines [7, 12] (Supplementary Fig. 4C–F and Supplementary Data 2 and 4).

**Fig. 3:**
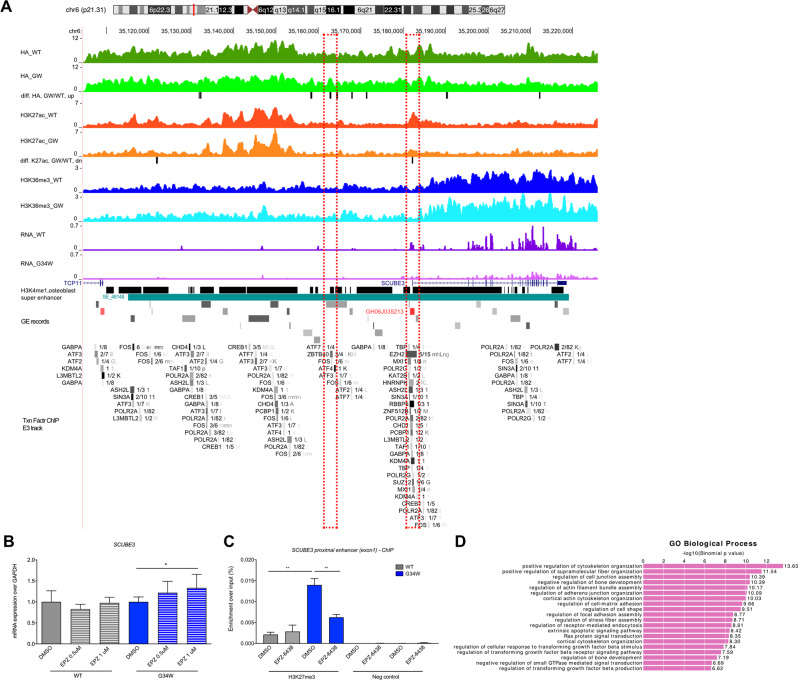
Changes in H3.3-HA, H3K27ac and H3K36me3 marks at the *SCUBE3* locus in hFOB lines. **A** H3.3-HA (top pair of tracks) H3K27ac modifications (3rd and 4th tracks), H3K36me3 modifications (5th and 6th tracks) at the *SCUBE3* locus and RNA expression (7th and 8th tracks) in H3.3^WT^ and H3.3^G34W^-hFOB. Differential peaks GWvsWT are shown with black bars. The super-enhancer SE_46148 (dbSUPER database) is reported in teal and the general enhancer GH06J035213 (reported in GE database) in red. TFs binding sites are reported from the Transcription Factor ChIP-seq Clusters (338 factors, 130 cell types) from ENCODE 3 track (Txn Factr ChIP E3). Tracks show average signal of replicates. Dashed red boxes identify the H3.3-HA (left) and the H3K27ac differential regions (right) described in the text. **B** Expression of *SCUBE3* by qPCR in H3.3^WT^ and H3.3^G34W^ hFOB following treatment with EPZ-6438 at the indicated concentrations. 3 experiments, 2 replicates per experiment. **C** ChIP-qPCR of H3.3^WT^ and H3.3^G34W^ hFOB following treatment with EPZ-6438 (1 μM) for H3K27me3 and negative control at the *SCUBE3* proximal enhancer region (Exon1). 1 experiment, 2 replicates. **D** Gene ontology analysis for the nearest genes of H3.3-GW/WT-up peaks bearing ZBTB40 motif. Data are mean ± SD. **B**: unpaired Student’s *t* test. **C**: 1-way Anova.

At the *SCUBE3* locus, we found that the promoter region contained both H3.3^WT^ and mutant H3.3^G34W^ (Fig. 3A). H3K36me3 distribution was not affected by H3.3^G34W^ whereas H3K27ac was significantly reduced compared to H3.3^WT^ cells in a region that overlaps with an osteoblast-specific super-enhancer, consistent with the observed reduction in *SCUBE3* mRNA expression (Fig. 3A).

Polycomb Repressive Complex 2 (PRC2) components such as zeste homologue 2 (EZH2) and other repressors (e.g., SIN3A) bind the proximal enhancer region around *SCUBE3* Exon 1, a previously reported area of accessible chromatin in GCT (Supplementary Fig. 4G), where H3K27ac is reduced in the presence of H3.3^G34W^ (Fig. 3A, see the Txn Factr ChIP E3 track). This prompted us to investigate if H3.3^G34W^-associated changes in H3K27ac at the *SCUBE3* promoter were dependent on PRC2-mediated regulation of H3K27me3, as described by others for G34R mutants [11, 21]. By treating H3.3^WT^ and H3.3^G34W^ hFOB cells with an EZH2 inhibitor (EPZ-6438) we found that *SCUBE3* expression was increased in H3.3^G34W^ but not in H3.3^WT^ cells (Fig. 3B). This was accompanied by higher H3K27me3 deposition at the *SCUBE3* proximal enhancer region in H3.3^G34W^ cells which was selectively reduced following treatment with the EZH2 inhibitor (Fig. 3C). These findings, along with data in the literature [12], suggest that H3.3^G34W^ may regulate the expression of *SCUBE3* by altering the balance between H3K27ac and H3K27me3 at its regulatory regions.

Although this *SCUBE3* proximal enhancer region did not show differences in H3.3-HA binding between H3.3^WT^ and H3.3^G34W^, other upstream regions presented differential enrichment. Of those, only one displayed overlap with known binding sites for transcription factors (TFs) or chromatin regulators, specifically FOS and ZBTB40 (Fig. 3A, see the Txn Factr ChIP E3 track), in a previously reported area of accessible chromatin in GCT (Supplementary Fig. 4G). FOS, which dimerises with JUN family members to form the AP-1 TF complex, is an acknowledged regulator of bone biology [22] and ZBTB40 has been implicated in regulating bone mineralisation in both humans and mice [23–28], suggesting that H3.3^G34W^ alters osteoblast differentiation by modulating the levels and/or binding of these TFs. It is noteworthy that gene ontology analysis of regions that show both H3.3^G34W^ gain and ZBTB40 binding are enriched in adhesion, migration and bone development pathways (Fig. 3D).

**Fig. 4:**
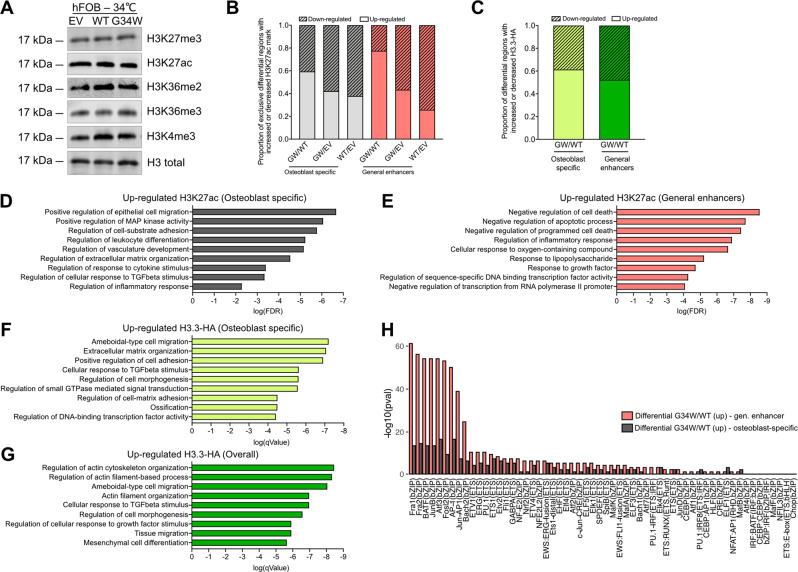
H3.3^G34W^ alters H3.3-HA, H3K27ac and H3K36me3 levels globally at enhancers of genes critical for osteoblasts. **A** Total levels of histone marks on histone preparations by western blot of undifferentiated (grown at 34 °C) hFOBs. **B** Proportion of exclusive differential peaks that show increased (up-regulated) or decreased (downregulated) H3K27ac marks from pairwise comparison among hFOBs, intersected with osteoblast-specific H3K4me1 genomic regions and general human enhancers. **C** Proportion of exclusive differential peaks that show increased (up-regulated) or decreased (downregulated) H3.3-HA binding comparing G34W/WT hFOBs, intersected with osteoblast-specific H3K4me1 genomic regions and general human enhancers. **D**, **G** Gene Ontology (GO) analysis of genes neighbouring differential H3K27ac peaks up-regulated exclusively in G34WvsWT intersected with (**D**) osteoblast-specific H3K4me1 regions or with (**E**) human general enhancers. Significance expressed as log(False Discovery Rate value) of top selected pathwas. Gene Ontology (GO) analysis of genes neighbouring differential H3.3-HA peaks up-regulated exclusively in G34WvsWT overall (**F**) or intersected with osteoblast-specific H3K4me1 regions (**G**). Significance expressed as log(qvalue) of top selected pathwas as reported in ClusterProfiler. **H** Significance of AP-1 and Ets TFs-motifs in differential H3K27ac peaks exclusively up-regulated in G34WvsWT, intersected with osteoblast-specific H3K4me1 regions (grey) and general human enhancers (coral).

In view of the changes in chromatin distribution at the *SCUBE3* locus, we next explored H3.3-HA, H3K27ac and H3K36me3 marks at the genome-wide level. Unlike previous reports on the effect of other oncohistones including H3.3^K27M^ and H3.3^K36M^ [20, 29, 30], western blot analyses did not reveal global alterations in H3K27ac and other histone marks in H3.3^G34W^ hFOB (Fig. 4A and Supplementary Fig. 5A). Analysis of the ChIP-Rx data revealed that a large number of regions displayed differential H3K27ac enrichment, with an overall increase in H3.3^G34W^ compared to WT and EV (Fig. 4B). Furthermore, the majority of differentially enriched peaks (GW/WT) overlapped with general human enhancers (12%) and mostly with reported osteoblast-specific H3K4me1 genomic regions (71%) (Supplementary Fig. 5B–G) which appeared skewed towards promoter regions (Supplementary Fig. 5H). We also identified many regions of H3.3-HA differential enrichment between H3.3^G34W^ and H3.3^WT^ (Fig. 4C and Supplementary Fig. 5I): the majority of differential peaks were in intronic and distant regions (Supplementary Fig. 5J) and did not significantly overlap with H3K27ac enriched regions (Supplementary Fig. 5K). At regions of differential enrichment, H3K36me3 was mostly gained in the presence of H3.3^G34W^ (Supplementary Fig. 6).

H3K36me3 differential regions were in close proximity to genes involved in the regulation of cell shape and bone metabolism (Supplementary Fig. 7A–C and Supplementary Data 5 and 6). Similarly, H3K27ac and H3.3-HA differentially enriched regions were located adjacent to genes involved in cell migration, cytoskeleton regulation, extracellular matrix organisation, response to cytokine stimulus as well as inflammation-related pathways and negative regulators of cell death (Fig. 4D–G, and Supplementary Fig. 7D–L and Supplementary Data 7–9). Analysis of binding motifs enriched at differential H3K27ac and H3.3-HA regions revealed predominantly AP-1/AP1-related and Ets/Ets-like motifs (Fig. 4H and Supplementary Fig. 8A–F) suggesting a role for AP1-related and Ets-like TFs, which are known to control bone homoeostasis and bone tumour development [22, 31]. AP-1/Ets TFs are therefore likely to be involved in H3.3^G34W^-dependent gene expression regulation at a global scale, although only FOS and ZBTB40 binding sites are identified at the SCUBE3 regulatory region (see Fig. 3A). Overall, these findings show that the presence of H3.3^G34W^ is associated with changes in genes involved in osteoblast biology, thereby explaining the histological phenotype of GCTs [7, 12].

Finally, H3K27ac was also altered (positively or negatively) at regions in the proximity of a large set of secreted factors, including members of the TGFβ pathway, to which SCUBE3 belongs, suggesting a wider role of H3.3^G34W^ in epigenetic regulation of the osteoblast secretome (Supplementary Fig. 8G).

### Reduced SCUBE3 expression contributes to increased osteoclast formation

**Fig. 5:**
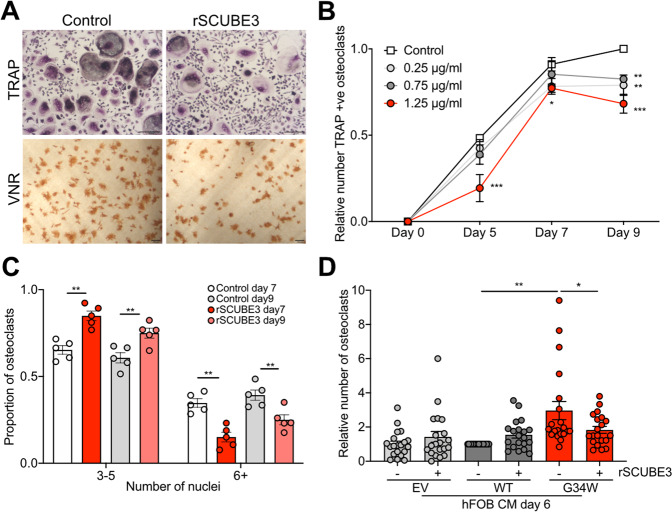
*SCUBE3* regulates osteoclast formation. **A** Representative photomicrographs on day 9 of osteoclasts generated from monocytes; treatment with rSCUBE3 results in fewer osteoclasts identifed by TRAP and vitronectin receptor (VNR) staining. **B** Dose-dependent inhibition of the total number of TRAP-positive osteoclasts by rSCUBE3; number relative to day 9 control cultures; 5 osteoclast preparations. **C** Quantification of number of nuclei per osteoclast following treatment with 1.25 μg/ml rSCUBE3, expressed as a proportion of OCs exhibiting 3–5 or ≥6 nuclei over the total number of OCs; 5 osteoclast preparations. **D** Number of osteoclasts generated in the presence of conditioned medium (CM) from differentiated (day 6) hFOBs in the presence or absence of 1.25 μg/ml rSCUBE3 (relative number to control H3.3^WT^ CM); 20 osteoclast preparations. Data represent the mean ± SEM. **B**, **C**: 2-way RM ANOVA. **D**: 1-way ANOVA.

Next, we assessed whether alterations in *SCUBE3* levels at least in part underlie the pro-osteoclastogenic effect of H3.3^G34W^-derived CM. Recombinant SCUBE3 (rSCUBE3) reduced the overall number and size of osteoclasts generated in vitro (Fig. 5A–C and Supplementary Fig. 9A–C), a finding supported by the altered expression of *OCSTAMP* and *DCSTAMP*, genes involved in osteoclast fusion (Supplementary Fig. 9D, E), without inducing cell death (Supplementary Fig. 9F–H). Finally, rSCUBE3 counteracted the osteoclastogenic effect of H3.3^G34W^-hFOB-CM (Fig. 5D). Together, these findings suggest that SCUBE3 is an inhibitor of osteoclastogenesis and its downregulation could explain the abundant giant osteoclasts in GCTs.

### Osteoclast-secreted SEMA4D provides the growth advantage to GCT

**Fig. 6:**
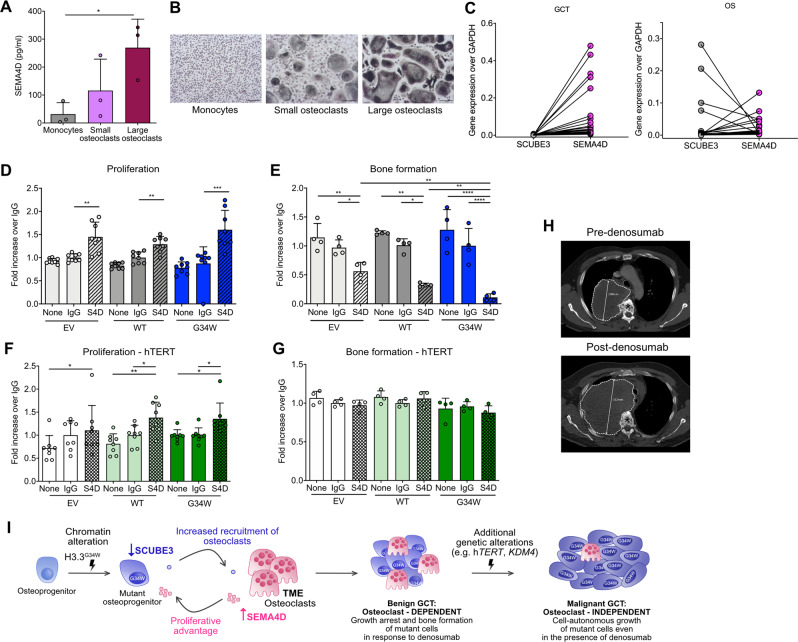
Benign and malignant GCTs respond differently to the TME. **A** ELISA for SEMA4D on the supernatant of human monocytes and small/large osteoclasts after 9 days of differentiation; 3 osteoclast preparations. **B** Representative images of osteoclasts (TRAP-positive cells) used in (**A**). **C** Expression of *SEMA4D* and *SCUBE3* by qPCR in GCTs (*n* = 32) and osteoblastic osteosarcoma (OS) samples with variable numbers of osteoclasts (*n* = 20). The GCT and OS samples represent a selection of those presented in Fig. 2D**;** levels of *SCUBE3* and *SEMA4D* are inversely related reflecting that osteoclast-poor bone-forming OSs have high levels of *SCUBE3* and low levels of *SEMA4D* whereas the opposite effect is observed in osteoclast-rich OSs. **D** Proliferation of hFOB transfectants in the presence of rSEMA4D, IgG control or mineralisation medium only (none) by Presto Blue assay after 7 days of proliferation at 34 °C; 2 experiments, 4 replicates each experiment. **E** Osteoimage assay of hFOB transfectants on day 6 of differentiation; 2 experiments, 2 replicates each experiment. **F** Proliferation of hTERT-hFOB transfectants by Presto Blue assay after 7 days of proliferation at 34 °C; 2 experiments, 4 replicates each experiment. **G** Osteoimage assay of hTERT-hFOBs on day 6 of differentiation; 2 experiments, 4 replicates each experiment. **H** Malignant GCT treated with denosumab: axial CT at T4-T5 vertebral level pre- and 3 months post-denosumab. In contrast to a conventional GCT, this tumour has grown (108–152 mm) (dotted outline) and mineralisation has not been induced by denosumab. Asterisk, vertebral body. **I** Proposed schema for GCT evolution. H3.3^G34W^-mutant osteoprogenitors express reduced levels of SCUBE3 resulting in increased formation of large osteoclasts, which secrete high levels of SEMA4D that block differentiation and promote proliferation of H3.3^G34W^-osteoprogenitors. The transition from benign to malignant GCT requires acquisition of at least one additional genomic alteration and malignant cells display cell-autonomous growth. Data are mean ± SD. **A**, **D**-**G**: 1-way ANOVA.

Next, we investigated whether osteoclast-derived factors contribute to the growth advantage of H3.3^G34W^ tumour cells. Whereas the majority of described osteoclast-derived molecules act by enhancing bone formation, Semaphorin 4D (SEMA4D) is one of the few known to suppress bone formation, and it is the only one that has also been implicated in tumour progression [14, 32, 33]. We demonstrate that SEMA4D is expressed by osteoclasts in GCT samples (Supplementary Fig. 9I) and that primary cultures of ‘large’ osteoclasts produced greater amounts of secreted SEMA4D compared with smaller osteoclasts (Fig. 6A, B). We also found that *SEMA4D* expression levels were inversely correlated to those of *SCUBE3* in both GCTs and osteosarcoma samples from patients: higher levels of *SEMA4D* were found in osteoclast-rich samples in which *SCUBE3* was low (Fig. 6C). Furthermore, treatment of hFOBs with recombinant SEMA4D (rSEMA4D) promoted proliferation equally in the three hFOB lines (Fig. 6D), whereas rSEMA4D reduced bone formation in H3.3^G34W^-hFOBs (Fig. 6E and Supplementary Fig. 9J–K). Together, these experiments argue that SEMA4D provides a growth advantage to the mutant cells in GCT by simultaneously enhancing their proliferation and blocking differentiation.

### Malignant GCTs do not rely on osteoclasts for growth

We next asked if the osteoclast-dependent growth advantage in conventional GCTs also occurs in malignant GCTs. We investigated the effect of the overexpression of *hTERT*, a common driver in malignant GCTs [3], on hFOBs. Irrespective of the H3.3 mutation status, *hTERT* induced a proliferative advantage (Supplementary Fig. 10A, B), but it did not alter cellular differentiation (Supplementary Fig. 10C). Treatment of hTERT-hFOBs with rSEMA4D increased proliferation of the three hFOB lines (Fig. 6F), but as the impact of *hTERT* alone was so great, the additive effect of rSEMA4D was minor (Supplementary Fig. 10B). However, rSEMA4D failed to block maturation of hTERT-cells (Fig. 6G and Supplementary Fig. 10D, E), a finding consistent with bone formation in malignant GCTs [34, 35]. These results suggest that the histone mutation is not required for sustaining growth following malignant transformation [3, 36] and that denosumab treatment would not curtail malignant disease. This concept is supported by the observation that denosumab treatment was ineffective in a patient with a malignant GCT harbouring a *hTERT* mutation (Fig. 6H and Supplementary Fig. 10F–H).

## Discussion

The TME is known to play an important role in neoplasia [37]. Here we show that the main genetic driver event, the H3.3^G34W^ mutation, in cells of the osteoblast lineage in benign GCTs exerts its growth-promoting effect in a non-cell autonomous manner through osteoclasts in the TME (Schema in Fig. 6I). Our evidence is supported by the clinical effect of denosumab, which results in growth arrest and maturation of the tumour cells into bone-forming cells. However, to date, the osteoclast-produced molecule(s) that promotes growth of the mutant osteoprogenitors has not been identified. Using human in vitro models, we show that SEMA4D provides a proliferative advantage to mutant osteoprogenitors. Our data also provide evidence that malignant GCTs would not benefit from treatment with denosumab: this assertion is made on our experimental evidence that mutant osteoprogenitors with a hTERT-mutated phenotype, characteristic of malignant GCTs [3], largely lose their dependency on osteoclasts for their proliferative advantage. Furthermore, our finding that SEMA4D does not block osteoblast differentiation in the presence of *hTERT* over-expression explains why malignant transformation of GCT can result in an osteosarcomatous histological phenotype [34].

Mechanistically, we find that H3.3^G34W^ in the absence of a proliferative advantage has a direct effect on osteoblast precursors by altering the expression of genes involved in extracellular matrix organisation and by inducing a block in osteogenic differentiation, a finding consistent with the histological phenotype of GCTs, also recently suggested by others [7, 12]. We demonstrate that H3.3^G34W^ modulates the profile of enhancers in the proximity of a large set of osteoblast-secreted factors, including *SCUBE3*, a molecule involved in the physiological bone growth and ossification process [18]. We provide evidence that reduction in expression of *SCUBE3* is a critical mechanism by which the conspicuous osteoclast population in GCTs occurs.

*H3-3A* and -*3B* are paralogous genes that transcribe the same product. Mutations in these genes occur in bone and paediatric brain tumours [2, 10] but the associated tumour type is largely specific for both the gene and mutation [38]. Our study shows that the H3.3^G34W^ mutation is associated with alterations of the epigenetic landscape of the mutant osteoprogenitors which notably comprise osteoblast-specific enhancers, including that of *SCUBE3*, thereby implicating this mutation in the pathogenesis of GCT specifically and not in other tumours. Our data suggest that H3.3^G34W^ may regulate the expression of *SCUBE3* by altering the balance between H3K27ac and H3K27me3 at its proximal enhancer region: these data are in keeping with recent work which shows a gain in H3K27me3 in G34R-mutated brain tumours [21] as well as in GCT cell lines [12]. Moreover, based on our observation of differential binding of H3.3^G34W^ at a site bound by the ZBTB40 TF, a known pro-osteogenesis factor in both mice and humans [23–28], we speculate that the mutant histone could affect the function and/or chromatin association of ZBTB40.

It remains to be established whether/how H3.3^G34W^ modulates H3K27ac levels. One possibility is that it interacts with the PRC2 complex at specific sites, thus leading to increased deposition of H3K27me3 and a subsequent decrease in H3K27ac. This would be an opposite effect to that of H3.3^K27M^, which inhibits H3K27me3 leading to increased H3K27ac at many enhancers [39]. However, we cannot exclude that the observed changes in H3K27ac are more a reflection of the cell of origin and/or differentiation stage at which this mutation arises [7]. This cell type-specific action of mutant H3.3 may also explain the different phenotype previously reported using other model systems [7, 11, 12, 40]. These two hypotheses are not mutually exclusive, as the mutant histone could also promote enhancer changes associated with earlier developmental stages, thus stimulating dedifferentiation of a more mature osteoblastic cell.

Collectively, our results provide mechanistic insights into the bidirectional communication between tumour cells and osteoclasts in benign GCT, a dependency which is largely lost on malignant transformation. Moreover, our work provides a starting point for the identification of similar processes in other mesenchymal neoplasms and more generally their potential roles in health and disease.

## Materials and methods

### Vector construction

pHIV-dTomato lentiviral vector (a gift from Bryan Welm, Addgene plasmid #21374) was used to transduce hFOB and iPSC. H3.3^WT^ and H3.3^G34W^ vectors were produced by linearising pHIV-dTomato using EcoRI to insert cDNA encoding C-terminal HA-tagged Drosophila *His3.3A* (a highly conserved ortholog of mammalian *H3-3A*) followed by the IRES and the dTomato sequences: GGA encoding for Glycine at position 34 was changed to TGG encoding for Tryptophan at position 34. The empty vector (EV) was used as a control. Lentiviral particles were produced as described previously [41]. hTERT was overexpressed in hFOB transfectants using the pCWX-UBI-hTert-PGK-BSD lentiviral vector (a gift from Patrick Salmon, Addgene plasmid #114316).

### Cell culture

Cell lines were expanded and differentiated according to the following protocols and cell authentication was regularly performed by Short Tandem Repeat fingerprinting (Culture Collections, Public Health England, UK) (Supplementary Table 1). Regular testing was also performed to ensure that the cell lines were mycoplasma-free using the EZ-PCR Mycoplasma Test Kit (K1-0210, Geneflow, Lichfield, Staffordshire, UK).

#### hFOB

hFOB1.19 ATCC® (CRL-11372™, ATCC, Manassas, VA, USA), a human fetal osteoblastic cell line immortalised using temperature-sensitive SV40 large T antigen which proliferates at the permissive temperature of 34 °C and undergoes osteoblastic differentiation at 39 °C [42], was grown in Dulbecco’s Modified Eagle Medium/Nutrient Mixture F-12 (21041025, Gibco Life Technologies, Thermo Fisher Scientific, Loughborough, Leicestershire, UK) supplemented with Fetal Bovine Serum (FBS) (F9665, Sigma Aldrich, St. Louis, MO, USA) to a final concentration of 10%, hereafter referred to as normal medium as described in the original publication [42]. This medium was supplemented with Geneticin® Selective Antibiotic (G418 Sulfate, 50 mg/mL, 10131035, Sigma Aldrich), which was added during maintenance culture (at 34 °C) but omitted when performing the osteogenic assays.

hFOB were infected with lentiviral particles an MOI of 10, incubated over night at 34 °C in the presence of the virus which was removed the next morning. The cells were expanded and FACS-sorted for dTomato. hFOB experiments were performed using cells derived from 4 independent infections. All experiments were performed using cells >90% dTomato positive.

To generate h*TERT*-expressing cells, hFOB-EV, -WT and –G34W cells were infected with lentiviral particles produced as described above, followed by antibiotic selection using Blasticidin S HCl (Thermo Scientific, A1113903, final concentration 5 µg/ml) and the surviving cells expanded for at least 3 passages before being used for functional assays.

To differentiate hFOB to mature osteoblasts (Supplementary Fig. 1), cells were seeded at a density of 0.4×10^6^ cells/well in 24 multiwell plates coated with collagen (Collagen, C3867, Sigma Aldrich), grown over night at 34 °C, then moved to 39 °C either in normal medium or in the presence of Osteoblast Mineralisation Medium (C-27020, Promocell, Heidelberg, Germany) [43] and grown for 6 days or until signs of mineralisation were evident (up to 12 days).

Conditioned medium (CM) of hFOBs grown in the absence of Geneticin was collected from cells seeded at a density of 0.4 × 10^6^ cells/well and grown overnight at 34 °C (day 0) or after 6 days at 39 °C and grown in mineralisation medium. Cell debris were removed by spinning at 2500 rpm for 5 min and CM was kept at −80 °C until needed.

#### Human iPSC-derived MSC

The viral-integration-free human iPSC line generated using cord blood-derived from CD34 + progenitors was obtained from Gibco™/Thermo Scientific (Cat. A18945) and grown in serum-free culture conditions according to the manufacturer’s instructions. Briefly, cells were cultured and expanded in 6-well plates coated with Geltrex™ LDEV-Free Reduced Growth Factor Basement Membrane Matrix (A1413202, Gibco Life Technologies) diluted 1:100 in DMEM (31966021, Gibco Life Technologies). Cells were maintained in Essential 8™ Flex Medium Kit (A2858501, Gibco Life Technologies) and passaged twice per week on reaching 80–90% confluency, using 0.5 mM EDTA in Dulbecco’s phosphate-buffered saline (14190250, Gibco Life Technologies); after which they were split (1:3 to 1:6) using trypsin. iPSC used in this study were between passages 40 and 75.

iPSC-derived MSC (Supplementary Fig. 2) were obtained by seeding iPSCs at a density of 50,000 cells/well in 12 well. 24 h later, mesoderm differentiation was induced by adding Cardiomyocyte Differentiation medium A (A29209-01, Gibco Life Technologies) for 48 h, after which iPSC-derived MSC were transduced. Cells were infected with lentiviral particles with an MOI of 15 using spinoculation (200–400 g at 34 °C for 30 min) and incubated overnight in the presence of virus, which was removed the following morning and replaced with fresh mineralisation medium or control MSC maintenance medium. iPSC-derived MSC cells were infected using fresh virus for each experiment and efficiency of infection was monitored by fluorescence microscopy. On the same day, osteoblast differentiation was induced by adding Mesenchymal Stem Cell Osteogenic Differentiation Medium (C-28013, Promocell) or Human Mesenchymal Stem Cell (hMSC) Osteogenic Differentiation Medium BulletKitTM (PT-3002, Lonza) according to manufacturer’s instructions. MSC used as control were passaged and maintained in MSCGM Mesenchymal Stem Cell Growth Medium BulletKitTM (PT-3001, Lonza). All steps were performed at 37 °C.

CM of osteoblasts differentiated from iPSC-derived MSC was collected from cells seeded at a density of 25000/well in a 24 multiwell plate after 8 or 15 days of osteoblasts differentiation. Cell debris were removed by spinning at 2500 rpm for 5 min and CM was kept at −80 °C until needed.

#### Human osteoclasts

Human osteoclasts were generated as described previously [44]: CD14+ monocytes were positively selected from the peripheral blood mononuclear cell component of leucocyte cones (NHS Blood and Transplant, UK) using CD14+ microbeads (130-050-201, Miltenyi Biotech, Surrey, UK). Monocytes were seeded onto dentine discs (elephant dentine; HM Revenue & Customs, Heathrow Airport, UK) or plastic dishes in α-MEM (without ribonucleosides/ deoxyribonucleosides; Lonza) containing 10% heat-inactivated FBS, 2 mM L-glutamine, 50 IU/ml penicillin and 50 μg/ml streptomycin sulphate. Osteoclastogenesis was induced by treatment with 25 ng/ml human M-CSF (216-MC, R&D Systems, Abingdon, UK) and 30 ng/ml RANKL (310-01, Peprotech, London, UK) every 3–4 days for 9 days. Small osteoclasts were generated for selected experiments using 3 ng/ml of RANKL, but otherwise were generated using 30 ng/ml of RANKL; monocytes were maintained in M-CSF in the absence of RANKL: CM for the ELISA was collected on day 9 of differentiation, 48 h after the last medium change. Each osteoclast preparation was generated from one leucocyte cone. All parameters analysed (number of osteoclasts, osteoclasts size etc) represent the average of 3 biological replicates with counts taken at 4 pre-defined fields of view per well. Use of leucocyte cones for osteoclast differentiation was approved by the London - Fulham Research Ethics Committee (11/H0711/7).

### Flow cytometry Activated Cell Sorting (FACS)

After transduction, hFOB cells were expanded to reach at least 2 × 10^6^ cells, dissociated into single cells and sorted using a BD FACS Aria Fusion Cell Sorter™ (Becton Dickinson, USA) running FACSDiva Software version 6. hFOB were bulk sorted to exclude DAPI + dead cells and to select dTomato-expressing cells (aiming for >99.9% positive cells). Positivity for dTomato of sorted cells was checked over time in different passages by Flow Cytometry, on an LSR Fortessa™ (Becton Dickinson, USA) running FACSDiva Software version 6 with 10^4^ events recorded for each sample.

### Osteogenic assays

#### Alizarin red staining (ARS) and quantification

Mineralised osteoblasts were fixed and stained according to the ‘Detection of Calcium Deposits (Mineralisation)’ Promocell protocol (PromoCell GmbH website). Briefly, fresh 2% (40 mM) ARS solution was prepared by adding 2 g of Alizarin (C.I.58005) to 100 mL of water and the pH was adjusted to 4.1–4.3 with 0.1% NH_4_OH. The solution was filtered and stored in the dark. Cells were gently washed with Phosphate Buffer Saline (PBS), fixed in neutral buffered formalin (10%) for 30 min, washed once with distilled water, incubated at room temperature in ARS solution for 45 min in the dark, washed 4 times with distilled water and kept in PBS. For the quantification, the stained cell monolayer was incubated at room temperature in 10% acetic acid for 30 min with shaking. Cells were collected using a cell scraper, vortexed for 30 s, heated at 85 °C for 10 min, incubated on ice for 5 min and then centrifugated at 20,000 × *g* for 15 min. Supernatant was transferred to a new tube and 10% ammonium hydroxide added to neutralise the acid. pH was checked in a small aliquot to ensure it fell within the range 4.1–4.5. The absorbance was read at 405 nm with a plate reader.

#### OsteoImage assay

Cells were seeded at a density of 100,000/well in collagen-coated 96 well plates, fixed after 12 days and stained according to the OsteoImage™ Mineralisation Assay Lonza kit (PA-1503, Lonza) protocol. Briefly, the cell monolayer was washed once in PBS, fixed in neutral buffered formalin (10%) for 30 min and rinsed in 1x OsteoImage™ Wash Buffer. OsteoImage™ staining reagent was added and incubated for 30 min at room temperature, protected from light. Cells were washed three times with washing buffer for 5 min. Fluorescence was read in a plate reader at excitation/emission wavelengths (492/520).

### Functional assays

#### Incucyte proliferation assay

hFOB were collected, counted and plated in TPP 96 well plates at 2500/well in at least 3 replicate wells per experiment. Cells were incubated at 34 °C or at 39 °C using an Incucyte Zoom® live cell imaging system (Essen BioScience, MI, USA). Images were taken every 2 h for 7 days and confluency was calculated using the Incucyte software.

#### Colorimetric viability assay

Cell viability was measured using Presto Blue Cell Viability Reagent (Cat A13262, Thermo Fisher Scientific, Loughborough, UK) according to the manufacturer’s instructions.

#### Edu proliferation assay

iPSC-derived MSC cells were differentiated in osteoblasts or maintained in MSC medium for 2 days, collected, stained and analysed using the Click-iT™ EdU Cell Proliferation Kit for Imaging, Alexa Fluor™ 647 dye (Cat. C10340, Thermo Scientific) according to manufacturer’s instructions. Experiments were performed on an LSR Fortessa™ (Becton Dickinson, USA) running FACSDiva Software version 6 with 10^4^ events recorded for each sample.

#### Apoptosis assay

iPSC-derived MSC cells were differentiated into osteoblasts for 2 days and collected. hFOB were grown at 34 °C until 70% confluency was reached and then collected. Apoptosis was determined by detecting phosphatidylserine using the APC-Annexin-V Apoptosis Detection Kit with PI (Biolegend, CA, USA). Briefly cells were harvested, washed once in PBS and 2 × 10^5^ cells resuspended in 250 µl of binding buffer containing 5 µL Annexin V-APC and 10 µl PI solution. Cells were incubated in the dark for 15 min before being analysed. Each assay was repeated 3 times, each with 3 replicates. Experiments were performed on an LSR Fortessa™ (Becton Dickinson, USA) running FACSDiva Software version 6 with 10^4^ events recorded for each sample.

#### Wound healing assay

A monolayer scratch assay was performed by seeding 30,000 cells/well in 24 well ImageLock plates (Essen Instruments, Cod. 4365) and incubating them for 3-4 days. hFOB were grown on plates coated with Collagen I solution (Sigma) at 34 °C. When cells were 95% confluent, wounds were created using the EssenBio wound maker and plates were scanned for 48 h using the Incucyte^TM^ FLR live cell imaging system (Essen BioScience, MI, USA). The system measures scratch closure in real time and automatically calculates the relative wound density within the initially empty area over a time course.

### Osteoclast formation, activity and survival assays

Tartrate-resistant acid phosphatase (TRAP) and the vitronectin receptor (CD51/61, VNR) are osteoclast markers used for the visualisation of mature osteoclasts [45]. TRAP staining was performed on formalin-fixed cells using naphthol AS-BI phosphate as a substrate, with reaction of the product with Fast Violet B salt. Multinucleated cells containing three or more nuclei were considered osteoclasts. VNR was detected on cells fixed in cold methanol by CD51/61 immunocytochemistry (clone 23C6, 1:400; Bio-Rad, Oxford, UK). Resorption tracks on dentine discs were visualised by staining with 0.5% toluidine blue under reflected light. The dentine slices were photographed, resorption tracks highlighted, and the resorbed area quantified using ImageJ. Terminal deoxynucleotidyl transferase dUTP nick end labelling (TUNEL) staining was performed using the In Situ Cell Death Detection Kit, POD (Sigma).

### Recombinant proteins, small molecules and conditioned medium (CM) treatments

hFOB were treated with the EZH2 inhibitor EPZ-6438 (A8221-APE, Stratech, Cambridge House, United Kingdom) at for 24 h at the indicated concentration; DMSO was used as control at the same percentage.

Effects of rSCUBE3 were investigated by treatment of osteoclast cultures with 0.25–1.25 μg/ml recombinant human rSCUBE3 protein (Cat # 7730-SC, R&D Systems, Abingdon, UK) in the presence of RANKL and M-CSF; all the reagents were replaced at each media change for osteoclasts. For control experiments, rSCUBE3 was denatured at 95 °C for 30 min.

Effects of osteoblast CM on osteoclasts were investigated by adding 10% CM generated from EV, WT or G34W hFOB or MSC-derived osteoblasts each time the medium was changed.

Effect of rSEMA4D was investigated by treating osteoblasts with recombinant Human Semaphorin 4D, Fc Tag 15 μg/ml (CDO-H5257, Acro Biosystem Newark, USA) or Recombinant Human IgG1 Fc 3 μg/ml (110-HG, R&D Systems, Minneapolis, MN, USA) as control. hFOB were seeded in collagen-coated 96 well plates (TPP) in 4 wells per genotype per condition at a density of 1×10^5^ cells/ well and kept at 34 °C for 24 h. For proliferation assay (colorimetric assay), the cells were kept in hFOB medium at 34 °C for 7 days. For bone formation assay the medium was replaced with Mineralisation medium (Promocell) and the plates moved to 39 °C for 12 days. Spent medium was replaced with fresh medium including fresh rSEMA4D/IgG every 4 days.

### RNA extraction and qPCR

Total RNA was extracted using miRNeasy Mini Kit (217004, Qiagen, Manchester, Lancashire, UK). Quantitative real-time PCR (qPCR) was performed as previously described [46]. FFPE and fresh frozen GCT tissue samples were processed for RNA extraction as described in Cottone at al. [47]. Primers used for qPCR are listed in Supplementary Table 2.

### Western blot and ELISAs

Western blots were performed as described in Scheipl et al. [46]. Histone extraction was prepared according to Abcam Histone Extraction protocol (Abcam website) [48]. Antibodies used for western blot are listed in Supplementary Table 3. Full-length uncropped original western are provided in Supplementary material.

#### ELISA

Protein quantification of CM from osteoclast cultures was performed using the Human SEMA4D (Semaphorin-4D) ELISA Kit (EH2196) (Wuhan Fine Biotech Co., China) according to manufacturer’s instructions.

### Immunohistochemistry (IHC) and Immunofluorescence (IF)

#### GCT samples

Tumour diagnoses were made using the WHO classification (WHO, 2020). Formalin-fixed paraffin-embedded (FFPE) samples were obtained from the archive of the Royal National Orthopaedic Hospital.

#### IHC

Hematoxylin and Eosin (H&E) staining and IHC were performed as described previously in [34] using the antibodies listed in Supplementary Table 3.

#### IF/Immunocytochemistry on iPSC

To confirm the expression of pluripotency markers in iPSC, the Pluripotent Stem Cell 4-Marker Immunocytochemistry Kit (Invitrogen™, Cat. A24881) was used according to manufacturer’s instructions. Images were acquired using an Axio Observer Z1 With Apotome.

Immunofluorescence for Ki67 was performed as described previously [41] using the antibody listed in Supplementary Table 3, on transduced MSC after differentiated to osteoblasts for 3 days. Quantification of Ki67 positive nuclei was performed analysing 10 images (20× magnification) per condition.

### RNA sequencing

hFOB grown at 34 °C for 15 days after transduction were collected in duplicate. Cells were lysed in Trizol and total RNA extracted using the Direct-zol kit (Zymo Research, CA, USA) including an on-column DNA digest. Poly(A) RNA was selected using the NEBNext Poly(A) mRNA Magnetic Isolation Module (New England Biolabs) and a first strand library prepared using NEBNext Ultra Directional RNA Library Prep Kit (New England Biolabs) and sequenced on a HiSeq 2500 (Illumina).

#### cDNA library construction and Illumina RNA-Seq

The cDNA libraries were constructed and sequenced by Source Bioscience, UK in accordance with the Illumina TruSeq RNA sample preparation guide v2 for Illumina paired-end multiplexed sequencing. In brief, the poly-A-mRNA in the extracted total RNA samples was purified using Illumina poly-T oligo-attached magnetic beads in two rounds of purification steps according to the manufacturer’s instruction. During the second step of poly-A RNA elution, the mRNA was fragmented and primed with random hexamers for cDNA synthesis. The first strand cDNA was synthesised from fragmented mRNA using reverse transcriptase and random primers. In a subsequent step, the RNA template was removed and a replacement was synthesised to construct double-stranded cDNA. After double-stranded cDNA synthesis, ends were repaired and an A-base was added to the blunt end fragments. Thereafter, Illumina indexing adapters were ligated according to the standard protocol for pooling of samples prior to sequencing and for subsequent identification of pooled samples in downstream analysis. The cDNA fragments that have adapter molecules on both ends were subjected to 15 rounds of PCR amplification. The concentration and size distribution of the synthesised cDNA libraries were confirmed using an Agilent BioAnalyzer 2100. The successfully amplified and indexed libraries were pooled and diluted to 10 nM prior to sequencing (two samples per lane). The molarity and size distribution were confirmed using an Agilent BioAnalyzer 2100. Finally, pooled samples were loaded at a concentration of 8 pM into each lane of an Illumina HiSeq 2000 flow cell v3 and sequenced with 100 bp paired-end reads.

#### RNA-seq data processing

The quality of the RNA-Seq data was examined using the package FastQC (http://www.bioinformatics.babraham.ac.uk/projects/fastqc/). RNAseq expression count estimates were made using kallisto software [49] together with the Ensembl GRCh38 (v99) transcript models. RNAseq count data were then imported using tximport [50] to the DESeq2 R package [51] for pre-processing, normalisation and statistical analysis. Multiple hypothesis adjustments used the independent hypothesis weighting method (IHW) [52]. Principal component analysis was performed on rlog transformed expression values [51].

### Chromatin immuno precipitation-sequencing normalised with an exogenous reference genome (ChIP-Rx)-sequencing and ChIP-qPCR

hFOB grown at 34 °C were collected in triplicate and washed once in cold PBS-5nM Na butyrate.

#### ChIP-Rx

A fixed ratio of *Drosophila* S2 cells (20% of hFOB cells) was spiked in prior to fixation to allow for exogenous normalisation. Cells were then fixed in 1% formaldehyde for 8 min prior to quenching with excess glycine. Fixed cells were resuspended on ice in wash buffer1 (10 mM Hepes pH7.5, 10 mM EDTA, 0.5 mM EGTA, 0.75% Triton X-100, all reagents from Sigma-Aldrich) for 5 min, rotating at 4 °C, centrifuged, and resuspended in wash buffer2 (10 mM Hepes pH 7.5, 200 mM NaCl, 1 mM EDTA, 0.5 mM EGTA, 0, all from Sigma-Aldrich) for 5 min rotating at 4 °C. Samples were then diluted with Lysis Buffer (150 mM Na-HCL, 25 mM Tris pH 7.5, 5 mM EDTA, 1% Triton X-100, 0.5% Deoxycholate, 0.2% SDS, all from Sigma-Aldrich) and sonicated on a Bioruptor Pico (Diagenode, Belgium) for 6–10 cycles of 30 s on/30 s off. After sonication, Triton was added to a final concentration of 1%. Sonication efficiency was checked by running a sample of de-crosslinked material on a 2% agarose gel. Equal amount of sonicated chromatin was incubated overnight rotating at 4 °C with the antibodies reported in Supplementary Table 3. Samples were incubated with protein A/G magnetic beads (Invitrogen) at 4 °C for 3 h. Beads were washed sequentially with buffer 1 (50 mM Tris, 500 mM NaCl, 1 mM EDTA, 1% Triton X-100, 0.1% sodium deoxycholate, 0.1% SDS, all from Sigma-Aldrich) three times, buffer 2 (20 mM Tris, 1 mM EDTA, 250 mM LiCl, 0.5% NP-40, 0.5% sodium deoxycholate, all from Sigma-Aldrich) three times and twice with TE buffer + 50 mM NaCl. DNA was eluted in buffer containing 50 mM Tris, 10 mM EDTA and 1% SDS before treatment with proteinase K and RNAse-A (both Thermo Fisher Scientific). DNA was purified with Qiaquick PCR purification Kit (Qiagen).

#### ChIP-Rx libraries construction and sequencing

Libraries were prepared using the NEBNext Ultra 2 DNA Library Preparation Kit (New England Biolabs, MA, USA) with AMPure® XP Beads (Beckman Coulter) and sequenced on a Nextseq500 (Illumina, CA, USA).

#### ChIP-Rx data processing

Raw data processing and alignment: low quality bases were trimmed, and adaptors were removed by Trimgalore with default parameters. Processed reads were aligned to hg19 and dm6 with “--no-unal” parameter. Alignment to hg19 was sorted, filtered to keep only normal chromosomes and indexed by Samtools. For H3K27ac and H3K36me3: initial scaling factors for each drosophila spiked-in IP samples were calculated following Niu et al. [53]. For each ChIP-type the initial scaling factors were adjusted by dividing initial scaling factors by the maximum initial scaling factor within the same ChIP antibody so that the maximum initial scaling factor was transformed to 1 and others accordingly. Filtered BAMs of IP samples were down-sampled by Picard with the final adjusted scaling factor. BigWig files were generated from scaled BAMs by bamCoverage with binSize = 1. Unique alignments from the filtered BAM files were subjected to peak calling by Homer using “histone” mode. For H3.3-HA ChIP: Reads were adaptor-trimmed and aligned to either dm6 or hg19 genome by bowtie2. The alignment was filtered by mapq value 10 and deduplicated by Picard. Number of mapq-filtered and de-duplicated aligned reads were used in calculation the preliminary scaling factors following the procedure in Niu et al. [53]. After preliminary scaling factor were obtained, the largest preliminary scale factor among samples to be compared (sf_max) was elevated to 1, and the preliminary scaling factors for the rest samples were divided by sf_max to obtain the final scaling factor. The filtered BAMs for human samples were downsampled by Picard using the final scaling factors.

Integration of histone modification peaks from experimental replicates and H3K4me1/general enhancer: for G34W and EV samples (each has two replicates) the intersected peak regions from two replicates were identified; for WT samples (three replicates) peak regions that were intersected by at least 2 out of 3 replicates were identified. Integrated H3K27ac peak set for each genotype derived from the last step was intersected with either H3K4me1 peak or general enhancer. Only intersected regions long than 50 bp were kept.

Differential peak identification: scaled BAM files were transformed to BED format by “bam2bed” from BEDOPS. diffReps was used to find differential peaks between all replicates of any two genotypes with window = 300 and meth = nb. Integration of differential H3K27ac peaks and H3K4me1/general enhancer: osteoblast H3K4me1 was downloaded from GEO (GSM733704 [54]) and general enhancer from FANTOM (https://fantom.gsc.riken.jp/5/datafiles/latest/extra/Enhancers/human_permissive_enhancers_phase_1_and_2.bed.gz). Both osteoblast-specific and general enhancer were intersected with differential peaks, and only intersected regions longer than 50 bp were kept. Exclusive differential peak (for differential H3K27ac they have been intersected with H3K4me1 or general enhancer first) for any pairwise comparison from three genotypes (WT, G34W, EV) were defined as differential peaks that do not overlap with differential peaks from the other two pairwise comparisons. Up- and down- exclusive differential peaks are distinguished according to diffReps output. Venn diagrams showing the overlapping among differential peak sets were generated using ChIPseeker R package. Differential binding between H3.3-G34W-HA and H3.3-WT-HA; integration with H3K4me1 and general enhancer: Differential binding between H3.3-G34W and H3.3-WT(r_gwWtUp) was identified directly by diffReps with settings “--pval 0.001 --frag 300 --window 250” and filtered by padj < 0.05 and log2FoldChange > 0. r_gwWtUp are then overlapped with either osteoblast H3K4me1 peaks or human general enhancer allowing minimum 50 bp overlapping.

Functional analysis: GREAT was used for functional analysis on up (increased histone modification)-/down(decreased histone modification)-/both- exclusive differential peak sets with default setting. Results in GO-Biological process, mouse phenotype single knockout and mouse phenotype were downloaded, and only terms shown in the default GREAT result were used for assembling the heatmap. The colour in the heatmap represents –log(hyperFDR). For H3.3-HA ChIP: the nearest genes were identified by annotatePeakInBatch function in ChIPpeakAnno R packge for r_gwWtUp and r_gwWtUp in H3K4me1 or general enhancer, and clusterProfiler was used for functional analysis.

Motif analysis: up (increased histone modification)-/down(decreased histone modification)-/both- exclusive differential peak sets were searched for enriched known motifs by Homer with size = 300. The heatmap for enriched motifs was generated by first collecting the union set of top 20 enriched motifs from each peak list, and p-values for the union motif were extracted from the motif discovery result for each peak list. If the *p* value was not found, it was assigned to 1. Motif analysis for H3.3-HA: r_gwWtUp was filtered by padj < 0.001 and followed by overlapping with H3K4me1, general enhancer or promoter with minimum overlapping 50 bp. Then Homer was used for motif discovery. ZBTB40 analysis: Probability matrix of ZBTB40 was downloaded from JASPAR. FIMO was used to detect occurrences of ZBTB0 in r_gwWtUp. r_gwWtUp containing ZBTB40 motif was fed into GREAT for functional analysis.

Genomic feature distribution: ChIPseeker was used to generate barplot from BED files that derived from “Integration of histone modification peaks from experimental replicates and H3K4me1/general enhancer” section.

Tag intensity profile: computeMatrix and plotProfile from deeptools were used to generate the tag density profile over genic and TSS from the scaled BigWig files with binSize = 10.

Principle component analysis: PCA plot was generated from scaled BAM files [55].

Differential H3K27ac peaks in TGF-beta signalling pathway: genes belonging to TGF-beta pathway were retrieved from MSigDB. The correspondence between differential H3K27ac peaks and nearby genes was identified by GREAT with default parameters. TGF signalling genes found in each differential peak list were assembled and represented as heatmap.

Functional analysis for differential H3K27ac peaks between G34W and WT overlapped with general enhancer bearing ETS-related motifs: DNA sequence of differential H3K27ac peaks (GW/WT,up) overlapped with general enhancer was retrieved by “bedtools getfasta” and saved as FASTA file. MEME-formatted motif matrixes for selected ETS-related motifs (ETV4, ETV1, GABP1, EHF, ERG, PU.1, ELF5, Fli1, ETS1, ETV2) were downloaded from JASPAR and catenated into one single file. FASTA file and the catenated motif file were supplied to FIMO to identify ETS-bearing peak. ETS-bearing peaks were then fed to GREAT for functional analysis with default settings.

The global H3K36me3 level for each sample was calculated as in Pathania et al. [56]:

$${{{{{{{\mathrm{Global}}}}}}}}\,{{{{{{{\mathrm{H}}}}}}}}3{{{{{{{\mathrm{K}}}}}}}}36{{{{{{{\mathrm{me}}}}}}}}3 = \frac{{\frac{{{{{{{{{\mathrm{Human}}}}}}}}\,{{{{{{{\mathrm{reads}}}}}}}}\,\left( {{{{{{{{\mathrm{H}}}}}}}}3{{{{{{{\mathrm{K}}}}}}}}36{{{{{{{\mathrm{me}}}}}}}}3} \right)}}{{{{{{{{{\mathrm{Drosophila}}}}}}}}\,{{{{{{{\mathrm{reads}}}}}}}}\,\left( {{{{{{{{\mathrm{H}}}}}}}}3{{{{{{{\mathrm{K}}}}}}}}36{{{{{{{\mathrm{me}}}}}}}}3} \right)}}}}{{\frac{{{{{{{{{\mathrm{Human}}}}}}}}\,{{{{{{{\mathrm{reads}}}}}}}}\,\left( {{{{{{{{\mathrm{input}}}}}}}}} \right)}}{{{{{{{{{\mathrm{Drosophila}}}}}}}}\,{{{{{{{\mathrm{reads}}}}}}}}\,\left( {{{{{{{{\mathrm{input}}}}}}}}} \right)}}}}$$

The following data are displayed in UCSC Genome Browser: Spike-in normalised IP signal from G34W and WT samples, BPM (Bins Per Million mapped reads) normalised BigWig for RNA-seq samples, differential H3K27ac peaks between G34W and WT, osteoblast H3K4me1 peaks from GEO (GSM733704(54)), super enhancer record for osteoblast from dbSUPER (http://asntech.org/dbsuper/index.php) and GeneHancer track from UCSC genome browser built-in.

### ChIP-qPCR

ChIP was perfomed as described above, without the addition of S2 cells spike-in chromatin. Pulled DNA was diluted 1:2 and analysed by qPCR as described above. Percentage of enrichment was calculated over the input (1%) and normalised for the H3-total enrichment for each sample. Neg control: ChIP performed without the addition of antibody. Primers and antibodies used are reported in Supplementary Tables 2 and 3.

### Statistics

Statistical parameters including the exact value of n, precision measures (mean ± SD) and statistical significance are reported in the Figures and Figure Legends. In figures, asterisk denote statistical significance with the following symbols: *p ≤ 0.05, **p ≤ 0.01, ***p ≤ 0.001, ****p ≤ 0.0001. Continuous variables were compared via unpaired or paired *t*-test. Grouped data were analysed using 1-way or 2-way ANOVA with Tukey’s or Dunnett’s multiple comparison as a post hoc test. Data are always mean ± SD. Statistical analysis was performed in GraphPad PRISM 8.0 (GraphPad Software, La Jolla, CA, USA).

### Terminology used to define replicates

“Experiment” refers to a replicate experiment that was performed multiple times (e.g., on different days, using different viral infections). “Replicates” refers to biological replicates within experiments (i.e., multiple wells exposed to treatment or differentiation conditions). “Technical repeats” of the same replicate (e.g., qPCR replicates for the same sample) have been averaged and not used as multiple values for statistical analysis.

## Supplementary information

Supplementary Material

Supplementary Data 1

Supplementary Data 2

Supplementary Data 3

Supplementary Data 4

Supplementary Data 5

Supplementary Data 6

Supplementary Data 7

Supplementary Data 8

Supplementary Data 9

Supplementary Data 10

Author contribution form

Checklist

## Data Availability

High-throughput data (RNA-seq and ChIP-Rx) of hFOB cells have been deposited in the National Center for Biotechnology Information GEO database under GEO accession number GSE152942.
