# Supplementary material for: Aberrant paracrine signalling for bone remodelling underlies the mutant histone-driven giant cell tumour of bone
Source: Cell Death Differ. 2022 Aug 3;29(12):2459–71. doi: 10.1038/s41418-022-01031-x (PMC9750984; doi:10.1038/s41418-022-01031-x)

Supplementary material for:

Aberrant paracrine signalling for bone remodelling underlies the mutant histone-driven giant cell tumour of bone

Lucia Cottone, Lorena Ligammari, Hang-Mao Lee, Helen J. Knowles, Stephen Henderson, Sara Bianco, Christopher Davies, Sandra Strauss, Fernanda Amary, Ana Paula Leite, Roberto Tirabosco, Kristian Haendler, Joachim L. Schultze, Javier Herrero, Paul O'Donnell, Agamemnon E. Grigoriadis, Paolo Salomoni and Adrienne M. Flanagan

Content

Supplementary Methods

Supplementary Figures and Legends 1-10

Supplementary Tables and Legends 1-4

Captions for Supplementary Data (Excel Spreadsheets) 1-9

Captions for Supplementary Data (PDF) 10

Captions for Supplementary Files (bed) uploaded on GEO (GSE152942)

References for Supplementary material

Uncropped full length original western blots

Supplementary Methods

Ratio of exogenous (drosophila) vs endogenous (human) H3.3 expression. cDNA of Drosophila H3.3A and H3.3B were incorporated into hg19 genome, and STAR index was built on this combined FASTA file. Adaptor-trimmed FASTQ files from RNA-seq of WT, G34W and EV were aligned to hybrid STAR index. GTF file of GRCh37 was modified by adding information of incorporated Drosophila H3.3A and H3.3B. This modified GTF file and BAMs aligned to hybrid STAR index were used in featureCounts to generate the count table (1). Normalisation was done by DESeq. Normalised expression of human and drosophila H3.3A, H3.3B and H3 total (HIST1H3A-J, HIST2H3A-C-D, H3F3A-B) were used in calculating ratio of H3.3 expression between drosophila and human.

Genome browser tracks of publicly available ATAC-seq data. ATAC-seq coverage data of GCT cells from H3.3^{WT} GCTs (UPI37-39) and H3.3^{G34W} GCTs (UPI34, UPI6-8) (2) was downloaded from ArrayExpress with accession number E-MTAB-9512.

Digital droplet PCR (ddPCR) on plasma samples. Blood samples were processed for extraction of cfDNA and DNA was analysed using ddPCR according to the protocol previously described (4). The presence of the canonical hTERT inactivating promoter mutation was detected using the BioRAD ddPCR EXD Assay TERT C228T_88, Has (dHsaEXD20945488, 12003908). The presence of the G34W mutation was detected using specific primers (G34W Fw 5' AAGCAACTGGCTACAAAA 3', G34W Rev 5' TGGATACATACAAGAGAGA 3') and probe (5' CCTCTACTGGAGGGGTGAAGAAA 3').

Supplementary Figures and Legends

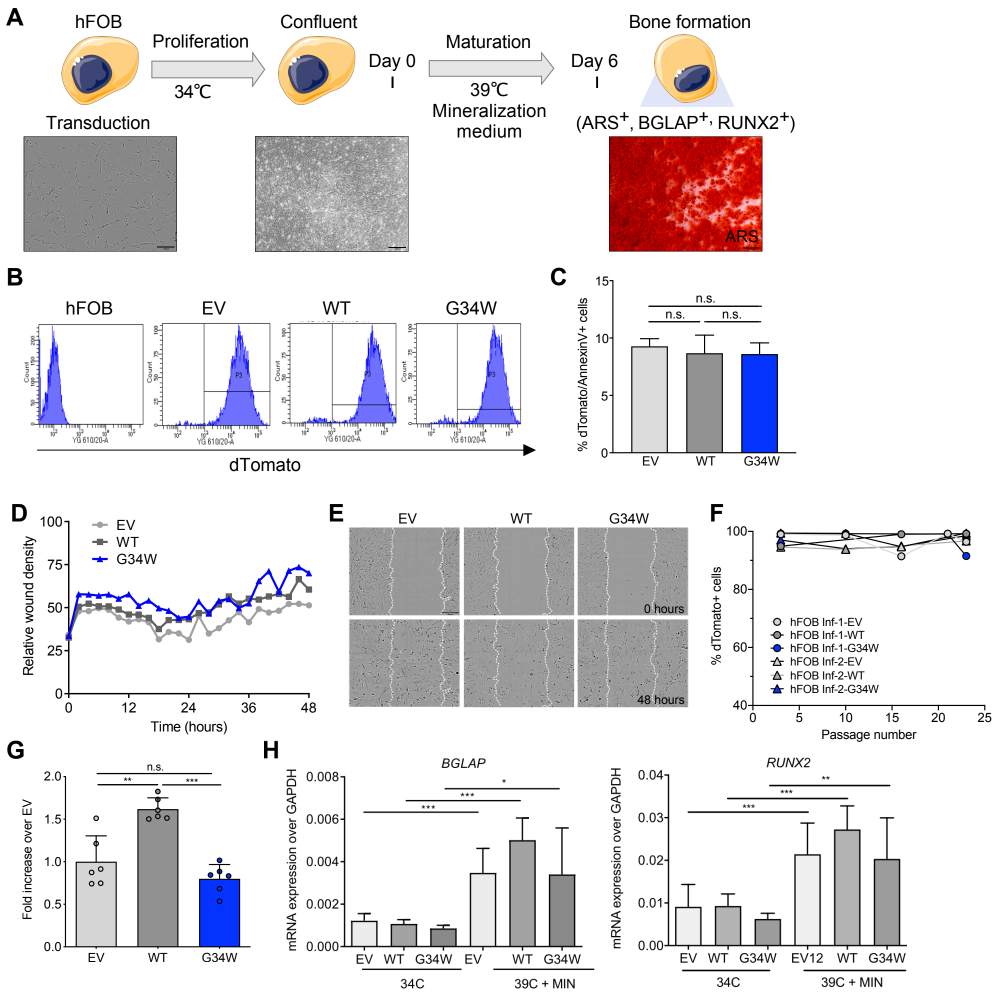

Supplementary Figure 1. Stable expression of H3.3^{G34W} in hFOB alters bone formation.

A. Schema of timeline of hFOB differentiation to mature osteoblasts and representative bright field images of hFOB showing bone formation by alizarin red staining (ARS). **B.** Percentage of dTomato-positive hFOBs transduced with H3.3^{WT}, H3.3^{G34W} and empty vector (EV) and assessed by flow cytometry after FACS sorting. **C.** Apoptosis of hFOB cultured at 34°C; 4 replicates. **D-E.** Wound healing assay: (D) quantification of the relative 'wound healing' assessed by Incucyte and (E) representative phase contrast images; 8 replicates, in 2 independent infections. **F.** Number of dTomato-positive hFOB in culture over time, in two independent infections (Inf-1 and Inf-2). All assays were performed using cells that were >90% dTomato-positive. **G.** Quantification of mineralisation of hFOB after 6 days of differentiation assessed by OsteoImage assay; 6 replicates. **H.** Gene expression of osteoblast genes *BGLAP* and *RUNX2* in hFOB at 34°C and after differentiation at 39°C in the presence of mineralisation medium (MIN) for 6 days; 2 independent experiments, 3 replicates per experiment. Data are mean+/-SD. C, G, H: 1-way ANOVA.

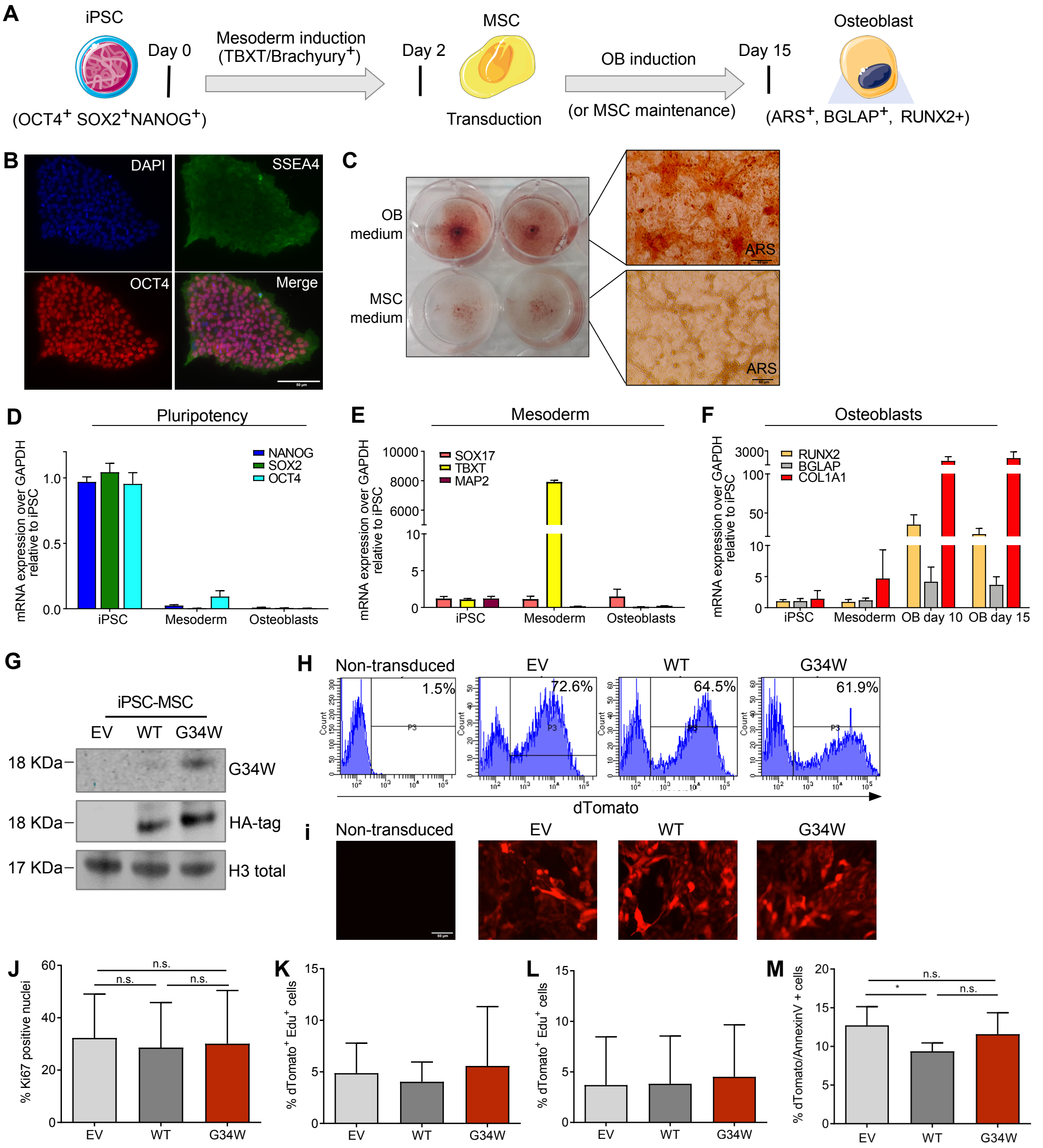

Supplementary Figure 2. Stable expression of H3.3^{G34W} in iPSC-derived MSCs.

A. Schematic timeline of differentiation of iPSCs to mesenchymal stem cells (MSC) via mesoderm induction, and then either maintained as MSC in MSC maintenance medium (MSC medium) or induced to mature into mineralised osteoblasts in osteoblast-inducing medium (OB medium). iPSCs were differentiated in MSC medium for 48 hours, transduced with lentiviral particles containing dTomato empty vector (EV), H3.3^{WT} or H3.3^{G34W} and then differentiated to osteoblasts. **B.** Analysis of the pluripotency markers OCT4 and SSEA4 expression in iPSCs using immunofluorescence. **C.** Bright field photomicrographs of calcium deposition detection by Alizarin Red staining (ARS) in non-transduced iPSCs on day 15 of differentiation to osteoblasts in OB medium, and in cells maintained in MSC maintenance medium. **D-F.** (D) Expression of genes of pluripotency (*NANOG*, *SOX2*, *OCT4*) is high in iPSC cells and suppressed during mesoderm induction and OB differentiation. (E) Expression of mesoderm-specific genes (*TBXT*) is induced upon mesoderm induction, whereas

expression of *SOX17* (marker of endoderm differentiation) and *MAP2* (marker of ectoderm differentiation) is suppressed. (F) Osteoblast-specific genes (*RUNX2*, *BGLAP*, *COL1A1*) are induced following osteoblast differentiation in a time-dependent manner; qPCR results, 3 replicates. **G.** Western blot: validation of H3.3^{WT}-HA and H3.3^{G34W}-HA overexpression on acid-extracted histone preparations of iPSC-derived MSCs. **H-I.** (H) Percentage of dTomato-positive iPSC-derived MSC as assessed by flow cytometry and (I) fluorescence microscopy 48 hours after transduction, 20X magnification. **J.** Number of Ki67-positive nuclei by immunofluoresence in iPSC-derived MSC differentiated osteoblasts (3 independent transductions, each with 2 replicates). **K-L.** EdU proliferation assay by flow cytometry: number of dTomato+EdU-positive cells on day 2 of (K) MSC maintenance in MSC maintenance medium and (L) osteoblast differentiation; 3 replicates. **M.** Apoptosis of iPSC-derived MSCs on day 4 of osteoblast differentiation assessed by AnnexinV-PI staining; 2 independent transductions, each with 3 replicates. Data are mean+/-SD. J-M: 1-way ANOVA.

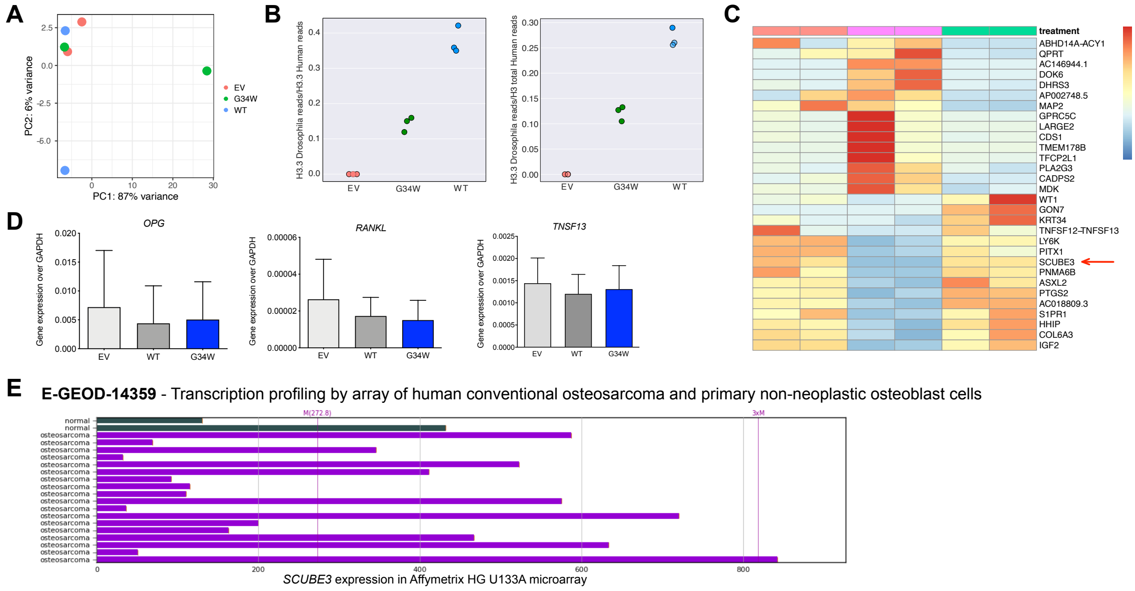

Supplementary Figure 3. RNA-sequencing of hFOB, expression of *SCUBE3.*

A. Principal component analysis (PCA) of RNA-seq data of hFOB performed on rlog transformed expression values. **B.** Ratio of normalised counts of drosophila H3.3 transcript on human H3.3 transcript and ratio of normalised counts of drosophila H3.3 transcript on human H3 total transcript from hFOB RNA-seq data. **C.** Heatmap shows statistically significant differences in gene expression, including *SCUBE3* indicated by the red arrow, between wild type (WT) and G34W hFOB samples (selection of genes, Independent Hypothesis Weighting (IHW)-adjusted p-value < 0.001). **D.** Expression of *OPG*, *RANKL* and *TNFSF13* by qPCR in hFOB transfectants cultured at 34ºC. 6 experiments, 2-3 replicates per experiment from 4 independent infections. **E.** Analysis of publicly available gene expression datasets reveal expression of *SCUBE3* in normal human osteoblast and human osteosarcoma samples (GSE14359(5)) as visualised on the BioGPS portal (6). Data are mean+/-SD. D: 1-way ANOVA, non-significant.

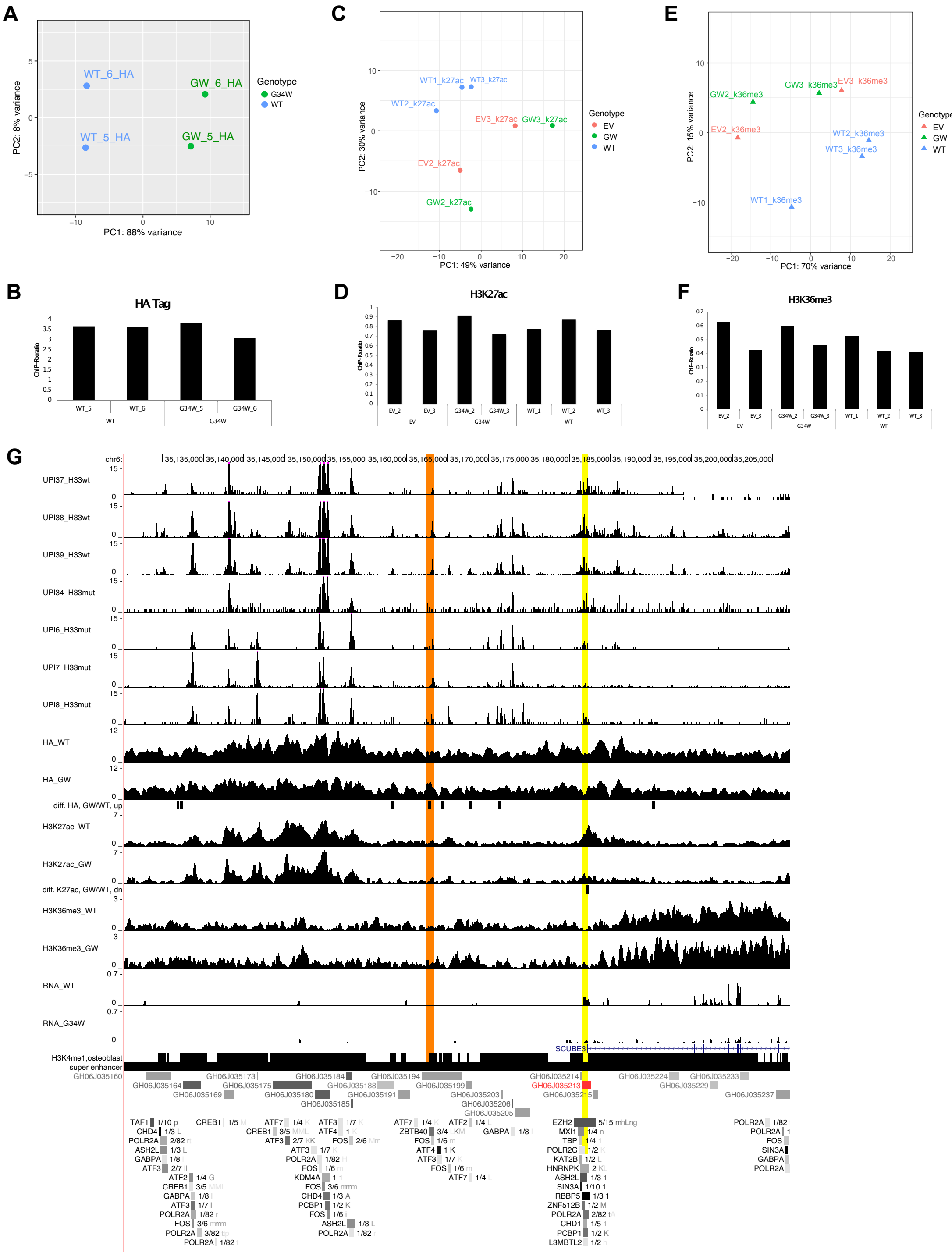

Supplementary Figure 4. H3.3-HA, H3K27ac and H3K36me3 Chromatin Immuno Precipitation-sequencing normalised with an exogenous reference genome (ChIP-Rx) of hFOB.

A. PCA of H3.3-HA ChIP-seq data of hFOB. **B.** Ratio of sequencing reads mapping to human and drosophila genome in H3.3-HA ChIP-seq, normalised by input. **C.** PCA of H3K27ac ChIP-seq data of hFOB. **D.** Ratio of sequencing reads mapping to human and drosophila genome in H3K27ac ChIP-seq, normalised by input. **E.** PCA of H3K36me3 ChIP-seq data of hFOB. **F.** Ratio of sequencing reads mapping to human and drosophila genome in H3K36me3 ChIP-seq, normalised by input. **G.** Genome browser tracks of publicly available

ATAC-seq data of GCT cells from H3.3^{WT} GCTs (UPI37-39) and H3.3^{G34W} GCTs (UPI34, UPI6-8) (2) and the hFOB H3K27ac and H3.3-HA ChIP-seq at the SCUBE3 regulatory region. Solid boxes identify the H3.3-HA differential region (orange) and the H3K27ac differential region (yellow) described in the text: both regions show peaks of chromatin accessibility which are smaller in H3.3^{G34W} cells compared to H3.3^{WT}.

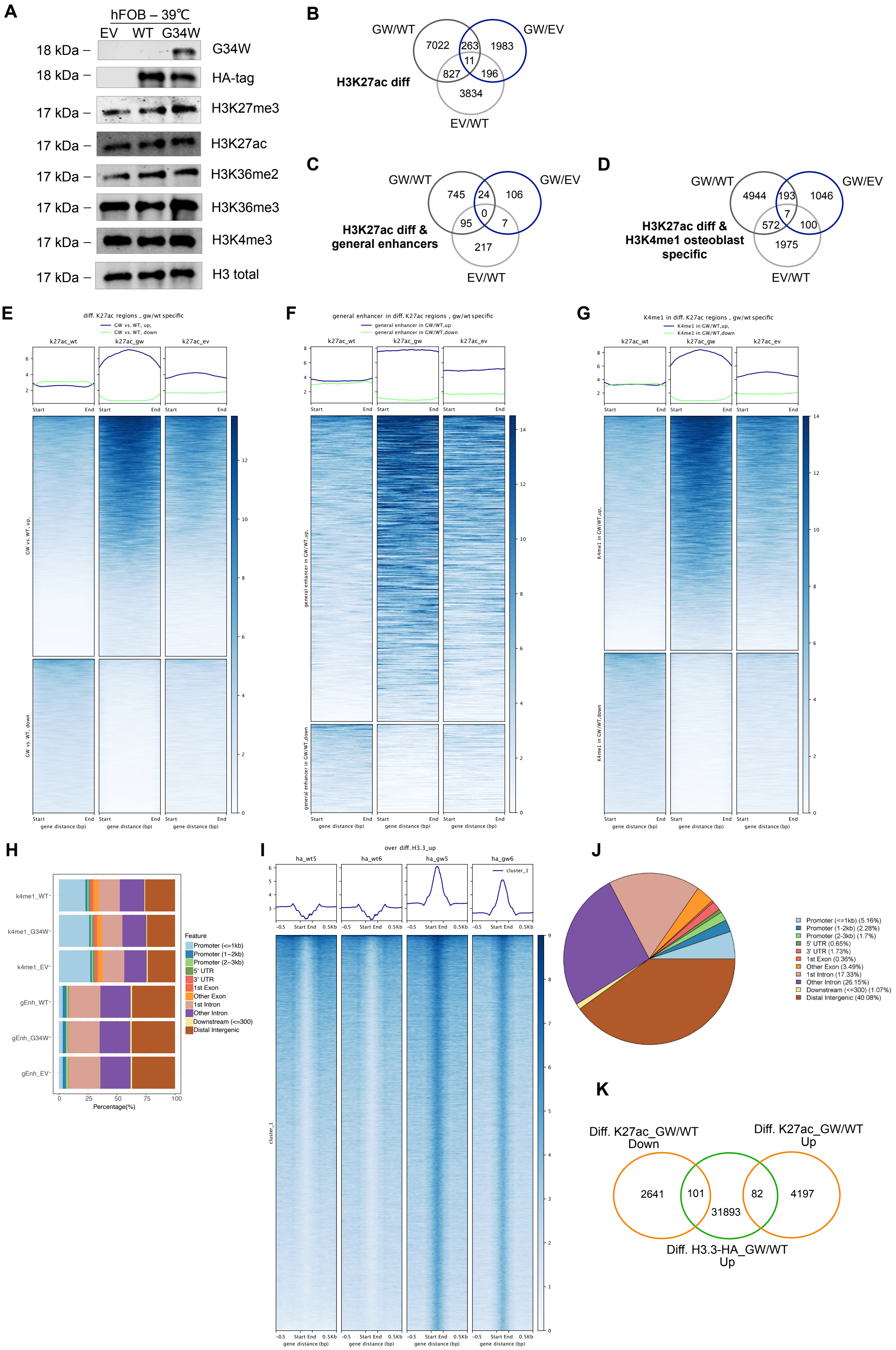
A
hFOB – 39℃
EV WT G34W
18 kDa – G34W
18 kDa – HA-tag
17 kDa – H3K27me3
17 kDa – H3K27ac
17 kDa – H3K36me2
17 kDa – H3K36me3
17 kDa – H3K4me3
17 kDa – H3 total
B
GW/WT
GW/EV
EV/WT
H3K27ac diff
7022
263
1983
11
827
196
3834
C
GW/WT
GW/EV
EV/WT
H3K27ac diff &
general enhancers
745
24
106
0
95
7
217
D
GW/WT
GW/EV
EV/WT
H3K27ac diff &
H3K4me1 osteoblast
specific
4944
193
1046
7
572
100
1975
E
diff. K27ac regions , gw/wt specific
GW vs. WT, up,
GW vs. WT, down
k27ac_wt
k27ac_gw
k27ac_ev
gene distance (bp)
F
general enhancer in diff. K27ac regions , gw/wt specific
general enhancer in GW/WT,up
general enhancer in GW/WT,down
k27ac_wt
k27ac_gw
k27ac_ev
gene distance (bp)
G
K4me1 in diff. K27ac regions , gw/wt specific
K4me1 in GW/WT,up,
K4me1 in GW/WT,down
k27ac_wt
k27ac_gw
k27ac_ev
gene distance (bp)
H
k4me1_WT
k4me1_G34W
k4me1_EV
gEnh_WT
gEnh_G34W
gEnh_EV
Percentage(%)
Feature
Promoter (<=1kb)
Promoter (1–2kb)
Promoter (2–3kb)
5' UTR
3' UTR
1st Exon
Other Exon
1st Intron
Other Intron
Downstream (<=300)
Distal Intergenic
I
over diff. H3.3_up
ha_wt5
ha_wt6
ha_gw5
ha_gw6
cluster_1
gene distance (bp)
J
Promoter (<=1kb) (5.16%)
Promoter (1-2kb) (2.28%)
Promoter (2-3kb) (1.7%)
5' UTR (0.65%)
3' UTR (1.73%)
1st Exon (0.36%)
Other Exon (3.49%)
1st Intron (17.33%)
Other Intron (26.15%)
Downstream (<=300) (1.07%)
Distal Intergenic (40.08%)
K
Diff. K27ac_GW/WT
Down
Diff. K27ac_GW/WT
Up
2641
101
31893
82
4197
Diff. H3.3-HA_GW/WT
Up

Supplementary Figure 5. H3K27ac and H3.3-HA ChIP-Rx of hFOB.

A. Validation of H3.3^{WT}-HA and H3.3^{G34W}-HA overexpression and total levels of histone marks (H3K27me3, H3K27ac, H3K36me2, H3K36me3, H3K4me3 and total H3) on acid-extracted histone preparations by western blot of hFOB after 3 days of differentiation at 39ºC. **B-D.** Venn diagram showing the number of differential H3K27ac peaks (B) overall, (C) intersected with general enhancers and (D) intersected with H3K4me1 osteoblast-specific genomic regions in hFOB. **E-G**. Average profiles and heatmaps of H3K27ac signal over differential regions (GW/WT-specific) overall (E), intersected with general enhancers (F) and intersected with H3K4me1 osteoblast-specific genomic regions (G) in hFOB. The signal represents the average of two or three biological replicates per condition. **H.** Distribution of genomic features of H3K27ac peaks intersected with H3K4me1 osteoblast-specific regions (top three bars) and intersected with general enhancers (bottom three bars) in hFOB. **I.** Average profiles and heatmaps of H3.3-HA signal over differential up-regulated regions (GW/WT-specific) overall in hFOB. The signal represents the average of two biological replicates per conditions. **J.** Distribution of genomic features of enriched H3.3-HA regions overall. **K.** Venn diagram showing the intersection of significantly differential regions among H3.3-GW/WT-up, H3K27ac-GW/WT-up and H3K27ac-GW/WT-down regulated regions.

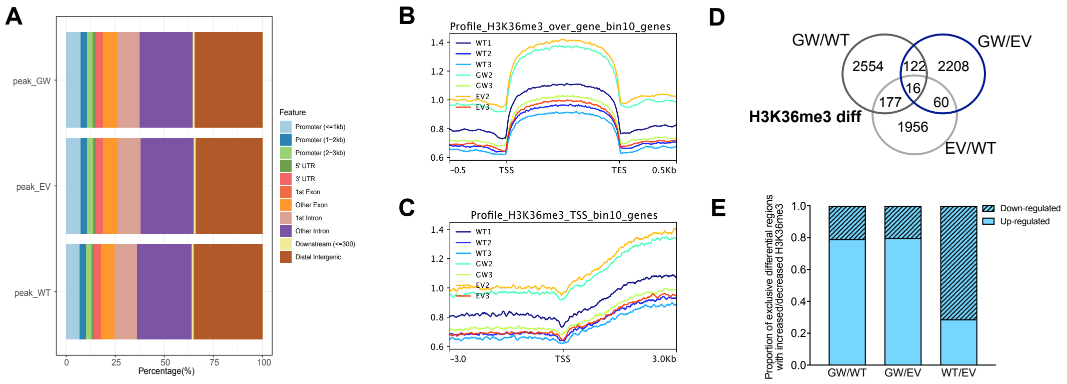

Supplementary Figure 6. H3K36me3 ChIP-Rx of hFOBs.

A. Distribution of genomic features of H3K36me3 peaks in hFOB. **B.** Positional profiles of H3K36me3 library around gene body. **C.** Positional profiles of H3K36me3 library around TSS. **D.** Venn diagram showing the number of differential H3K36me3 peaks in hFOB. **E.** Proportion of exclusive differential peaks showing increased (up-regulated) or decreased (down-regulated) H3K36me3 marks from pairwise comparison among H3.3^{G34W} (GW), H3.3^{WT} (WT) and EV.

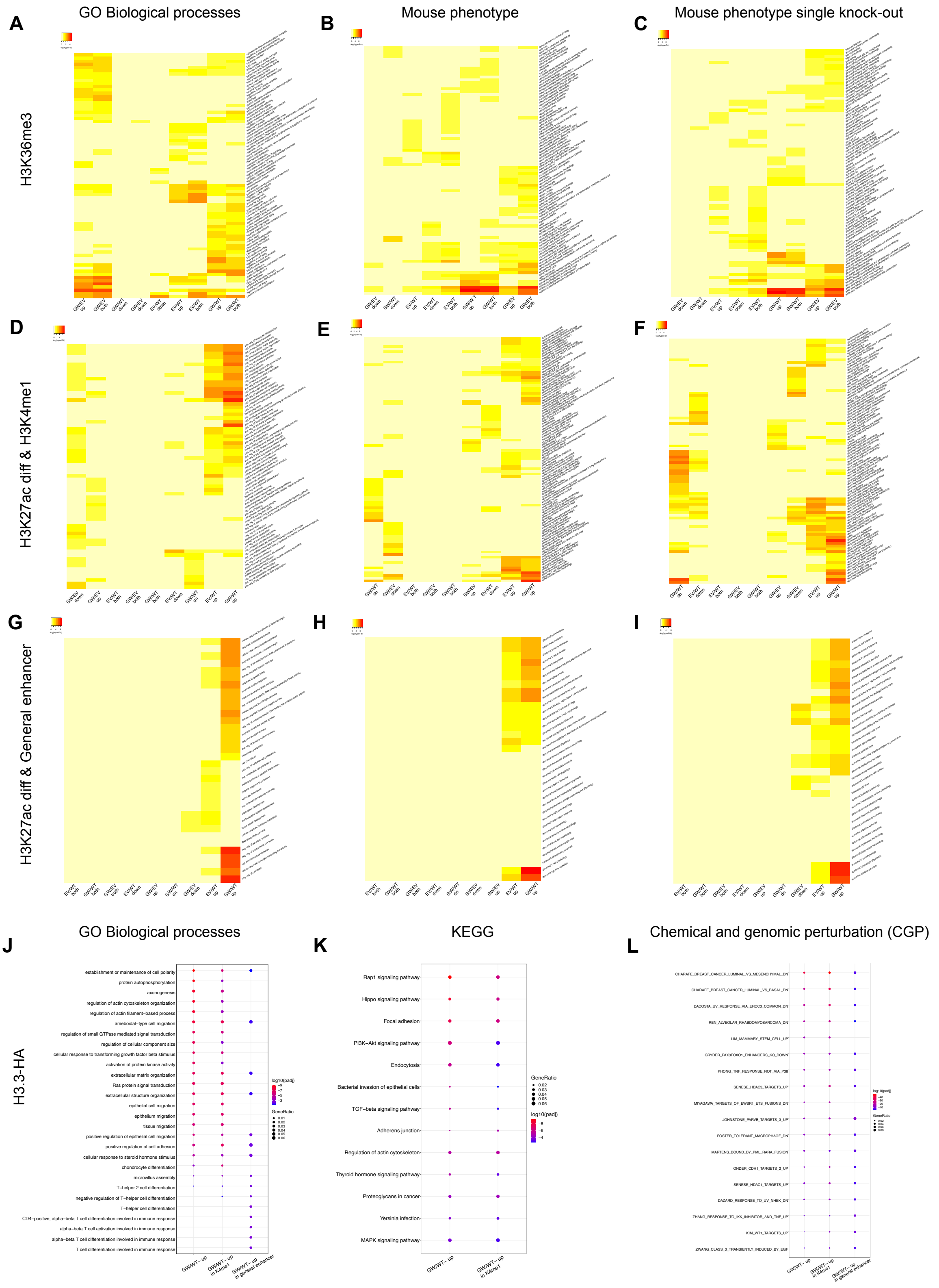

Supplementary Figure 7. H3.3-HA, H3K27ac and H3K36me3 ChIP-Rx of hFOB: functional analysis.

A-C. Heatmaps of functional analysis for GO biological processes, mouse phenotype and mouse phenotype single knock-out for exclusive differential up-regulated or down-regulated H3K36me3 peaks. **D-I.** Heatmaps of functional analysis for GO biological processes, mouse phenotype and mouse phenotype single knock-out for exclusive differential H3K27ac peaks intersected with (D-F) osteoblast-specific H3K4me1 genomic regions and (G-I) general enhancers in hFOB. **J-L.** Dot plots for functional analysis of Gene Ontology (GO) biological processes, Kyoto Encyclopedia of Genes and Genomes (KEGG) and chemical and genomic perturbation (CGP) for H3.3-HA differential up-regulated in G34W versus WT (GW/WT) hFOBs, overall, overlapping with H3K4me1 and overlapping with general enhancers.

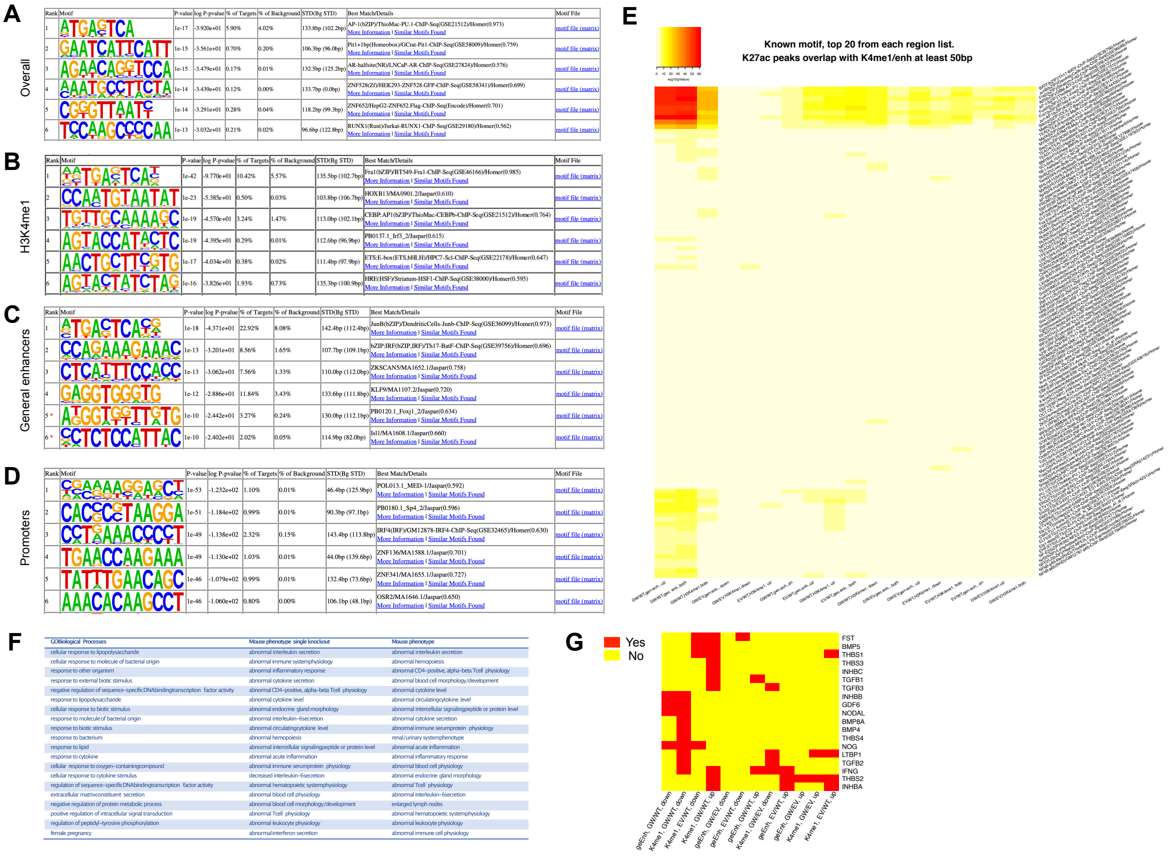

Supplementary Figure 8. Transcription factor (TF) motif analysis for H3.3-HA, H3K27ac and H3K36me3 ChIP-Rx of hFOB.

A-D. Top 6 enriched motifs in GW/WT up-regulated regions overall (A), overlapping with H3K4me1 genomic regions (B), general enhancers (C) and promoters (D). The full list of motifs is provided in **Supplementary Material 10**. **E.** Heatmap of TF binding motifs in exclusive differential H3K27ac peaks intersected with either osteoblast-specific H3K4me1 genomic regions or general enhancers in hFOB. **F.** Functional analysis of Ets and Ets-like motifs-containing differential H3K27ac peaks up-regulated exclusively in G34W vs WT intersected with general enhancers: Ets domains were sited close to genes involved in the immune response, cytokines and interleukin secretion. **G.** Heatmap of secreted genes in the TGF−beta family with exclusive differential H3K27ac peaks in hFOB.

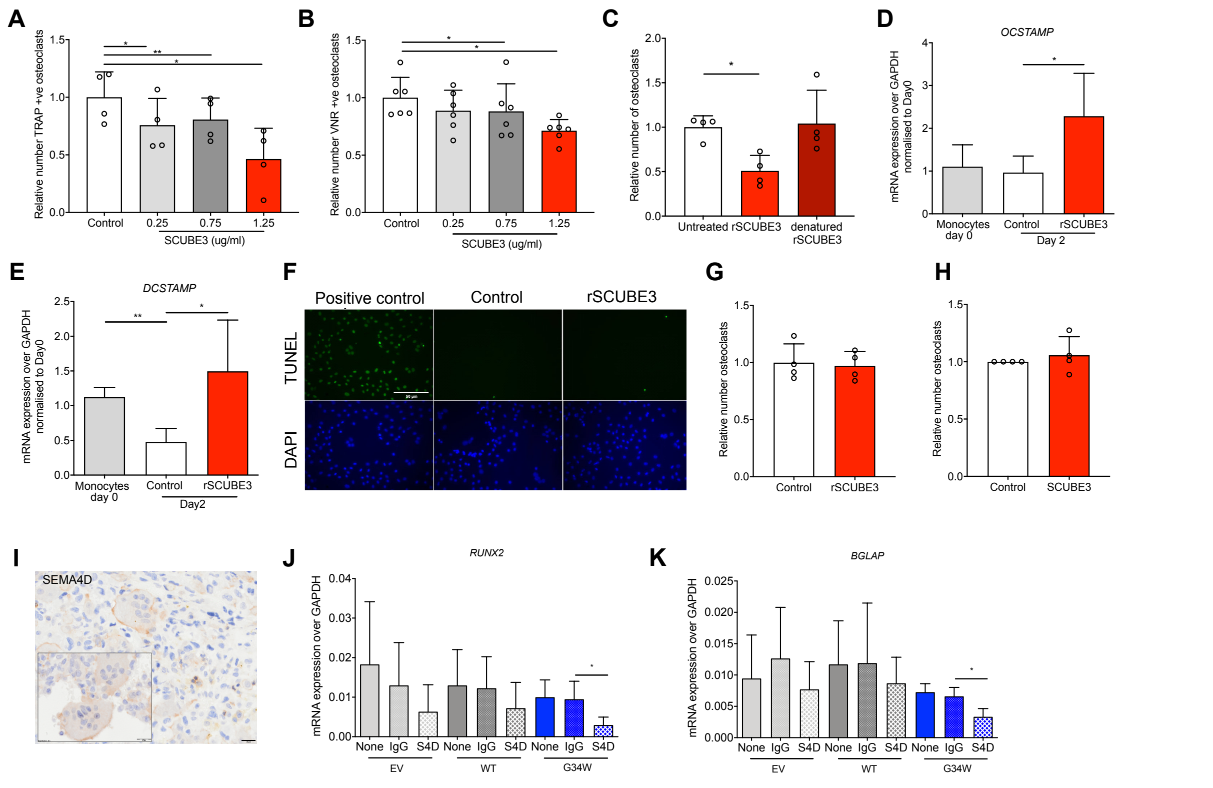

Supplementary Figure 9. Role of SCUBE3 and SEMA4D in benign GCT.

A-B. Number of (A) TRAP-positive and (B) VNR-positive osteoclasts on day 9 of differentiation in the presence of increasing concentrations of rSCUBE3; 4 and 6 osteoclasts preparations respectively. **C.** Treatment with 1.25 µg/ml rSCUBE3 denatured by heat inactivation does not reduce the number of osteoclasts formed; 4 osteoclast preparations. **D-E.** rSCUBE3 alters the expression of genes involved in osteoclast fusion, *OCSTAMP* and *DCSTAMP*, assessed by qPCR, in untreated monocytes (day 0) and in osteoclasts differentiated from monocytes for 2 days in the absence or presence of 1.25 µg/ml rSCUBE3; 4 osteoclast preparations. **F.** Fluorescent images of TUNEL staining of osteoclasts at day 9 of differentiation, following differentiation in the presence of 1.25 µg/ml rSCUBE3; 3 osteoclast preparations **G-H.** Survival of mature (day 8) osteoclasts treated for 48 hours with 1.25 µg/ml rSCUBE3 or vehicle: number of TRAP- (G) and VNR- (H) positive osteoclasts at day 10; 4 preparations. **I.** Photomicrograph of GCT showing immunoreactivity of SEMA4D depicting the membrane of osteoclasts; 40X magnification. **J-K.** Gene expression by qPCR of osteoblast genes *BGLAP* and *RUNX2* in hFOB transfectants in the presence of rSEMA4D, IgG control or mineralization medium only (none) for 6 days; 2 experiments, 2 replicates per experiment. Data are mean+/-SD. A-E, J-K: 1-way ANOVA. G-H: unpaired t-test, non significant.

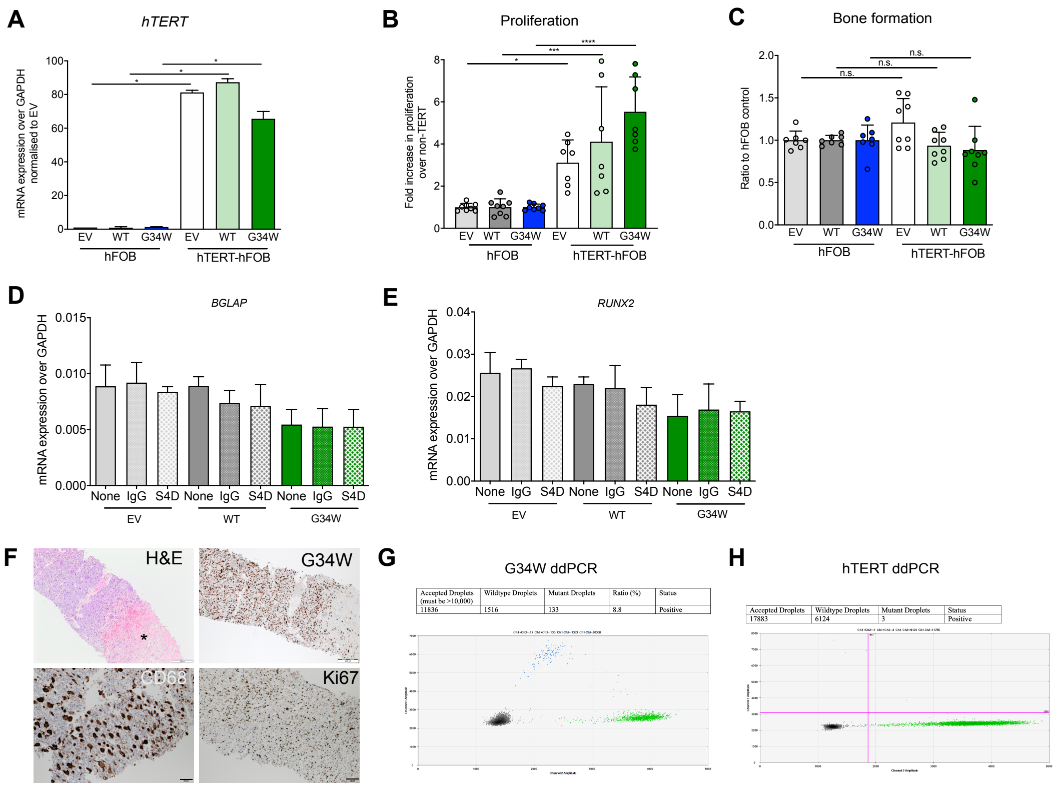

Supplementary Figure 10. Role of SCUBE3 and SEMA4D in malignant GCT.

A. qPCR analysis of expression of h*TERT* in hFOB transfectants before (hFOB) and after over-expression of hTERT (hTERT-hFOB); 2 replicates per condition. **B.** Proliferation of hFOB and hTERT-hFOB transfectants as assessed by the Presto Blue assay after 7 days of proliferation at 34°C; 2 experiments, 7-8 replicates in total per condition. **C.** Bone formation in hFOB and hTERT-hFOB transfectants on day 6 of differentiation as assessed by the Osteoimage assay; 2 experiments, 7-8 replicates in total per condition. **D-E.** Gene expression by qPCR of osteoblast differentiation genes *BGLAP* and *RUNX2* in hTERT-hFOB in the presence of rSEMA4D, IgG control or mineralisation medium only (none) for 6 days; 2 experiments, 2 replicates per experiment. **F.** Photomicrograph of an osteoclast-rich G34W-mutant malignant GCT pre-treatment (histology of tumour shown in **Figure 6H**), showing necrosis (asterisk), presence of CD68-immunoreactive osteoclasts and atypical highly proliferative H3.3^{G34W}-mutant stromal cells (G34W, Ki67). **G-H.** Post-denosumab treatment (of tumour in **Figure 6H**) showing detection of G34W (G) and hTERT promoter (H) mutations in the circulating cell-free tumour DNA (cfDNA) using digital droplet PCR. This demonstrates that growth of the G34W-mutant tumour cells is independent of osteoclasts. Mutant G34W molecules in cfDNA have not been detected in benign GCT other than when

associated with fracture (4). Data are mean+/-SD. A-C: 1-way ANOVA. D-E: 1-way ANOVA, non-significant.

Supplementary Tables

Supplementary Table 1. Cell line authentication by STR (short tandem repeat) profiling of hFOB used in this study (report June 2020).

		UCL profiles		Database profiles	
Cell line	Marker	Allele 1	Allele 2	Allele 1	Allele 2
**hFOB**	AMEL	X	X	X	X
	CSF1PO	10	13	10	13
	D13S317	11	12	11	12
	D16S539	9	13	9	13
	D18S51	10	17	10	17
	D21S11	29	32.2	29	32.2
	D3S1358	17	18	17	18
	D5S818	11	12	11	12
	D7S820	8	10	8	10
	D8S1179	10	14	10	14
	FGA	19	22	19	22
	Penta D	9	13	9	13
	Penta E	8	11	8	11
	TH01	7	9.3	7	9.3
	TPOX	11	11	11	11
	vWA	16	18	16	18

Supplementary Table 2. List of primers used for qPCR.

	Gene	Primer sequences (5'-3')
**Control gene**	*GAPDH*_Fw	GAAGGTGAAGGTCGGAGTCA
	*GAPDH*_Rev	GAAGATGGTGATGGGATTTC
**Pluripotency markers**	*NANOG*_Fw	AACTGGCCGAAGAATAGCAA
	*NANOG*_Rev	TGCACCAGGTCTGAGTGTTC
	*OCT4*_F w	CCTCACTTCACTGCACTTGTA
	*OCT4*_Rev	CAGGTTTTCTTTCCCTAGCT
	*SOX2*_Fw	ATGTCCCAGCACTACCAGAG
	*SOX2*_Rev	GCACCCCTCCCATTTCCC
**Endoderm layer**	*SOX17*_FW	TGTTCAGAGATTTGTTTCCCATAG
	*SOX17*_RV	ACACACCCAGGACAACATTTC
**Ectoderm layer**	*MAP2*-Fw	CCACCTGAGATTAAGGATCA
	MAP2-Rev	GGCTTACTTTGCTTCTCTGA
**Mesoderm layer**	*TBXT*_Fw	CCCGTCTCCTTCAGCAAAGTC
	*TBXT*_Rev	TGGATTCGAGGCTCATACTTATGC
**Osteoblast genes**	*BGLAP*_Fw	AATCCGGACTGTGACGAGTT
	*BGLAP*_Rev	GGCAAGGGGAAGAGGAAAGA
	*RUNX2*_Fw	CTGTGGTTACTGTCATGGCG
	*RUNX2*_Rev	AGGTAGCTACTTGGGGAGGA
	*COL1A1*_Fw	GTGCTAAAGGTGCCAATGGT
	*COL1A1*_Rev	CTCCTCGCTTTCCTTCCTCT
	*SCUBE*_Fw	GTATGCTGGTTGTCGCTGAG
	*SCUBE*_Rev	GGTTGTGTGCATGACTGTGT
	OPG_Fw	AGTGTCTATACTGCAGCCCC
	OPG_Rev	CAGCTTGCACCACTCCAAAT
	RANKL_Fw	TAATGCCACCGACATCCCAT
	RANKL_Rev	ATGTTGGAGATCTTGGCCCA
	TNFSF13_Fw	AAGAAGTATGCCCTCCCACC
	TNFSF13_Rev	ATGGAAGACACCTGCGCTAT
**hTERT**	*hTERT*_Fw	GCCGATTGTGAACATGGACTACG
	*hTERT*_Rev	GCTCGTAGTTGAGCACGCTGAA
**GCT human samples**	*SCUBE3*_70bp_Fw	GTGATGACACAGAGCAGGGT
	*SCUBE3*_70bp_Rev	CACAGGTCTCGATGCATGTCT
	SEMA4D_Fw	GGCGTGGAGAGTTTTGTGTT
	SEMA4D_Rev	CTGAGGTTGTCTGCACGATG
	*GAPDH*_78pb_Fw	CATACCAGGAAATGAGCTTGACAA
	*GAPDH*_78pb_Rev	ACACCCACTCCTCCACCTTTG

**ChIP-qPCR**	*SCUBE3_Promot_Fw*	GCTCTGCCTGCTTGTCCT
	SCUBE3_Promot_Rev	CCCCTCCTTCCTTACCTTGC

Supplementary Table 3. List of antibodies used (western blot, IF, IHC, ChIP).

Protein	Use	kDa	Brand	Cat. Number	Species	Clonality	Dilution
H3 Total	WB	17	Abcam	Ab 1791	Rabbit	Polyclonal	1:1000
H3K27me3	WB	17	Millipore	07-449	Rabbit	Polyclonal	1:2000
H3K36me3	WB, ChIP	17	Abcam	Ab9050	Rabbit	Polyclonal	1:1000
H3K36me2	WB	17	CST	9758	Rabbit	Polyclonal	1:1000
H3K27ac	WB, ChIP	17	Abcam	Ab4729	Rabbit	Polyclonal	1:1000
H3K4me3	WB	17	Abcam	Ab8580	Rabbit	Polyclonal	1:1000
B-actin	WB	42	Sigma	A5441	Mouse	Monoclonal	1:5000
HA-tag	WB, ChIP		Abcam	Ab9110	Rabbit	Polyclonal	1:1000
H3.3 G34W	WB, IHC		RevMab Biosciences	31-1145-00	Rabbit	Clone RM263	1:250
Anti-mouse Alexa Fluor 594	IF		Thermo Scientific		Mouse		1:500
Goat anti-Rabbit Alexa Fluor 488	IF		Thermo Scientific	A-11034	Rabbit	Polyclonal	1:500
IRDye 800CW Goat anti-Rabbit	WB		Thermo Scientific	SA5-35571	Rabbit	Polyclonal	1:5000
IRDye 680CW Goat anti-Mouse	WB		Thermo Scientific	SA5-35518	Mouse	Polyclonal	1:5000
Ki67	IF		Abcam	Ab16667	Rabbit	Clone SP6	1:200
Ki67	IHC		Leica	PA0118	Mouse	MM1	Ready to use
CD68	IHC		Leica	PA0273	Mouse	514H12	Ready to use

WB, Western blot.

Captions for Supplementary Data

Supplementary Data 1 (Excel spreadsheet):
Supplementary_Data_1_hFOB_EV-WT_G34W_RNAseq_DEG. List of DEGs with a p-adj value <0.05 identified by RNA-sequencing of hFOB (**Figure 2**): comparisons of G34W versus WT, G34W versus EV, EV versus WT.

Supplementary Data 2 (Excel spreadsheet):

Cottone_Supplemental_Data_2_Peak_Count_HA_H3K27ac_H3K36me3. Number of differential peaks among G34W, WT and EV, separating up- and down-regions for H3.3-HA, H3K27ac and H3K36me3 ChIP.

Supplementary Data 3 (Excel spreadsheet):

Cottone_Supplemental_Data_3_ScaleFactor_Count_HA. Read counts for calculating scaling factors for the H3.3-HA ChIP-Rx.

Supplementary Data 4 (Excel spreadsheet):

Cottone_Supplemental_Data_4_ScaleFactor_Count_H3K27ac_H3K36me3. Read counts for calculating scaling factors for the H3K27ac and H3K36me3 ChIP-Rx.

Supplementary Data 5 (Excel spreadsheet):
Supplementary_Data_5_H3K36me3_Pathway_Analysis_GWvsWT_all. List of Gene Ontology (GOBP), mouse phenotype (MousePh) and mouse phenotype single knock out (MousePhSKO) analysis of differential H3K36me3 peaks up- or down-regulated in G34W vs WT.

Supplementary Data 6 (Excel spreadsheet):
Supplementary_Data_6_MatrixHmap_diffH3K36me3_spec_ALL. –log10(HyperFdrQ) values for heatmaps of functional analysis for GO biological processes (GOBP), mouse phenotype (MousePh) and mouse phenotype single knock-out (MousePhSKO) for exclusive differential H3K36me3 peaks in hFOB.

Supplementary Data 7 (Excel spreadsheet):
Supplementary_Data_7_H3K27ac_Pathway_Analysis_GWvsWT_all. List of Gene Ontology (GOBP), mouse phenotype (MousePh) and mouse phenotype single knock out (MousePhSKO) analysis of differential H3K27ac peaks up- or down-regulated in G34W vs WT, intersected with osteoblast-specific H3K4me1 enhancers and general enhancers.

Supplementary Data 8 (Excel spreadsheet):
Supplementary_Data_8_MatrixHmap_diffH3K27ac_spec_ALL. –log10(HyperFdrQ) values for heatmaps of functional analysis for GO biological processes (GOBP), mouse phenotype (MousePh) and mouse phenotype single knock-out (MousePhSKO) for exclusive differential H3K27ac peaks intersected with H3K4me1 osteoblast-specific genomic regions and general enhancers in hFOB.

Supplementary Data 9 (Excel spreadsheet): Cottone_Supplemental_Data_9_H33-HA_Pathway_Analysis_GWvsWT_all. List of GO, KEGG and CGP pathways of differential H3.3-HA peaks up-regulated in G34W vs WT, overall, intersected with osteoblast-specific H3K4me1 enhancers and general enhancers.

Supplementary Data 10 (PDF):

Cottone_Supplementary_Data_10_MotifAnalysis_HA. Enriched motifs in GW/WT-up regions overall, overlapping with H3K4me1, overlapping with general enhancers and overlapping with promoters.

Captions for Files uploaded on GEO (GSE152942)

The following files contain the differential regions between GW/WT, GW/EV and EV/WT for H3.3-HA, H3K27ac and H3K36me3 ChIP. Differential regions have been split into up-/down-regions. These 18 region files correspond to regions represented by 9 circles in the Venn diagrams in **Supplementary Figure 5 B-D**. The up-/down-regions in each circle were separated into individual files.

A_20211116_k27ac_gwVsWt_gw1Except_Up.bed

B_20211116_k27ac_gwVsWt_gw1Except_Down.bed

C_20211116_k27ac_gwVsEv_gw1Except_Up.bed

D_20211116_k27ac_gwVsEv_gw1Except_Down.bed

E_20200427_k27ac_evVsWt_Up.bed

F_20200427_k27ac_evVsWt_Down.bed

G_20200601_k4me1_intersect_k27ac_gwWt_up_50bp.bed

H_20200601_k4me1_intersect_k27ac_gwWt_dn_50bp.bed

I_20200601_k4me1_intersect_k27ac_gwEv_up_50bp.bed

J_20200601_k4me1_intersect_k27ac_gwEv_dn_50bp.bed

K_20200601_k4me1_intersect_k27ac_evWt_up_50bp.bed

L_20200601_k4me1_intersect_k27ac_evWt_dn_50bp.bed

M_20200601_gEnh_intersect_k27ac_gwWt_up_50bp.bed

N_20200601_gEnh_intersect_k27ac_gwWt_dn_50bp.bed

O_20200601_gEnh_intersect_k27ac_gwEv_up_50bp.bed

P_20200601_gEnh_intersect_k27ac_gwEv_dn_50bp.bed

Q_20200531_interRegion_genEnh_k27ac_evWtUp_50bp.bed

R_20200531_interRegion_genEnh_k27ac_evWtDn_50bp.bed

The following files contain the differential regions (up-/down-separated and GW/WT-specific) of H3K4me1 that overlap with differential H3K27ac and general enhancer that overlap with differential H3K27ac. These six files are equivalent to the GW/WT-specific regions in **Supplementary Figure 5 B-D** after splitting up-/down-regions.

A_20211103_k27ac_gwWt_gw1Excl_specific_up.bed

B_20211103_k27ac_gwWt_gw1Excl_specific_dn.bed

C_20211103_k4me1_k27ac_gwWt_gw1Excl_specific_up.bed

D_20211103_k4me1_k27ac_gwWt_gw1Excl_specific_dn.bed

E_20211103_genh_k27ac_gwWt_gw1Excl_specific_up.bed

F_20211103_genh_k27ac_gwWt_gw1Excl_specific_dn.bed

The following file contains regions where H3.3-G34W is more enriched than H3.3-WT detected in H3.3-HA ChIP, all regions.

A_20211025_h33_gwVsWt_noRep7_pnasParam_q10RmdupBiop_pj005_f0_up_osteo202103.bed

The following files contain G34W-enriched regions overlapped with general enhancer, H3K4me1 regions and promoters detected in detected in H3.3-HA ChIP,

O_20211105_h33_gwVsWt_noRep7_pnasParam_q10RmdupBiop_pj005_f0_up_osteo202103_inGenhMinOl50.bed

P_20211105_h33_gwVsWt_noRep7_pnasParam_q10RmdupBiop_pj005_f0_up_osteo202103_inK4me1MinOl50.bed

Q_20211105_h33_gwVsWt_noRep7_pnasParam_q10RmdupBiop_pj005_f0_up_osteo202103_inPromoterMinOl50.bed

References for Supplementary material

1. Liao Y, Smyth GK, Shi W. The Subread aligner: Fast, accurate and scalable read mapping by seed-and-vote. Nucleic Acids Res. 2013;41(10).

2. Lutsik P, Baude A, Mancarella D, Öz S, Kühn A, Toth R, et al. Globally altered epigenetic landscape and delayed osteogenic differentiation in H3.3-G34W-mutant giant cell tumor of bone. Nat Commun. 2020;11(1):1–37.

3. Ramírez F, Dündar F, Diehl S, Grüning BA, Manke T. DeepTools: A flexible platform for exploring deep-sequencing data. Nucleic Acids Res. 2014;42(W1):187–91.

4. Gutteridge A, Rathbone VM, Gibbons R, Bi M, Archard N, Davies KEJ, et al. Digital PCR analysis of circulating tumor DNA: a biomarker for chondrosarcoma diagnosis, prognostication, and residual disease detection. Cancer Med. 2017;6(10):2194–202.

5. Fritsche-Guenther R, Noske A, Ungethüm U, Kuban RJ, Schlag PM, Tunn PU, et al. De novo expression of EphA2 in osteosarcoma modulates activation of the mitogenic signalling pathway. Histopathology. 2010;57(6):836–50.

6. Wu C, Orozco C, Boyer J, Leglise M, Goodale J, Batalov S, et al. BioGPS: An extensible and customizable portal for querying and organizing gene annotation resources. Genome Biol. 2009;10(11).

Uncropped full length original western blots

Figure 1D

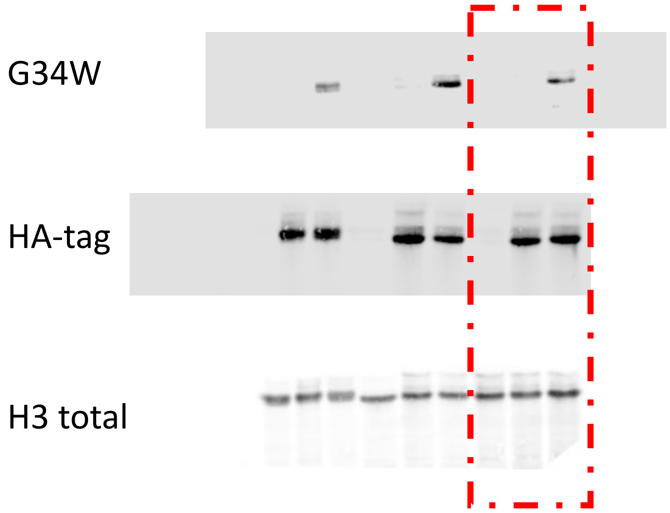

Figure 4A

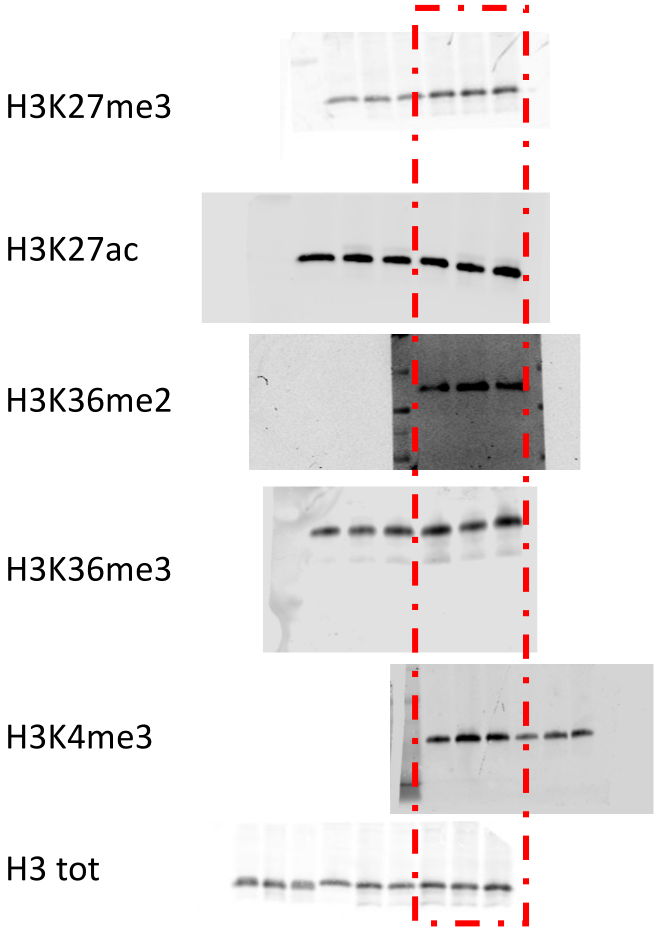

Supplementary Figure 5A

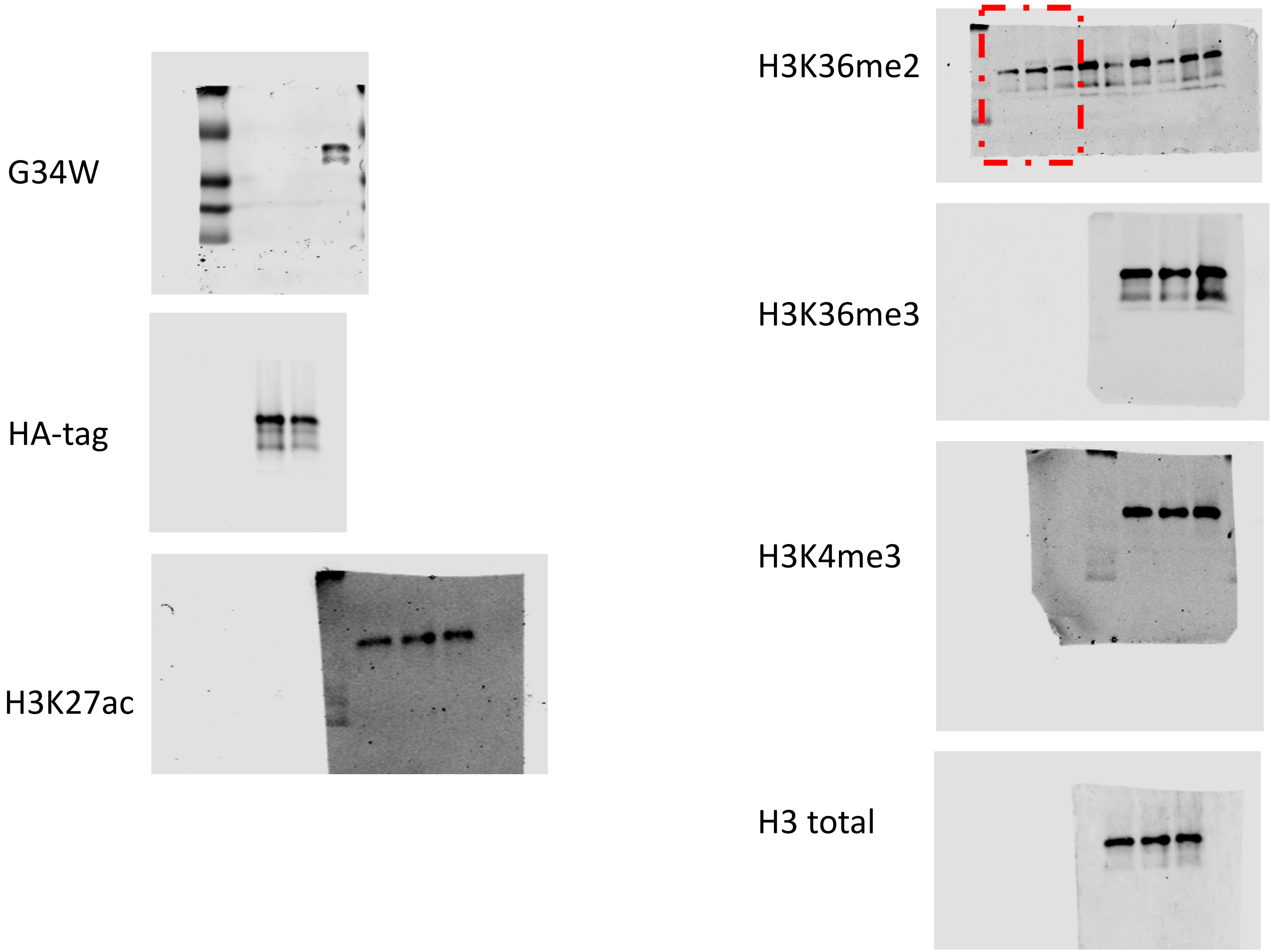

Supplement: Supplementary file 1 — Supplementary Material [file 41418_2022_1031_MOESM1_ESM.pdf]
