# Supplementary material for: Aberrant paracrine signalling for bone remodelling underlies the mutant histone-driven giant cell tumour of bone
Source: Cell Death Differ. 2022 Aug 3;29(12):2459–71. doi: 10.1038/s41418-022-01031-x (PMC9750984; doi:10.1038/s41418-022-01031-x)
Supplement: Supplementary file 11 — Supplementary Data 10 [file 41418_2022_1031_MOESM11_ESM.pdf]

Homer *de novo* Motif Results (motif/20211105_h33_gwVsWt_noRep7_pnasParam_q10RmdupBiop_pj001_f0_up_osteo202103_zbtb40InDb/)

Known Motif Enrichment Results
Gene Ontology Enrichment Results
If Homer is having trouble matching a motif to a known motif, try copy/pasting the matrix file into STAMP
More information on motif finding results: HOMER | Description of Results | Tips
Total target sequences = 9064
Total background sequences = 41240
* - possible false positive

Rank	Motif	P-value	log P-pvalue	% of Targets	% of Background	STD(Bg STD)	Best Match/Details	Motif File
1	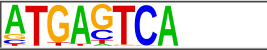	1e-17	-3.920e+01	5.90%	4.02%	133.8bp (102.2bp)	AP-1(bZIP)/ThioMac-PU.1-ChIP-Seq(GSE21512)/Homer(0.973)<br>More Information | Similar Motifs Found	motif file (matrix)
2	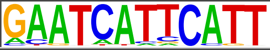	1e-15	-3.561e+01	0.70%	0.20%	106.3bp (96.0bp)	Pit1+1bp(Homeobox)/GCrat-Pit1-ChIP-Seq(GSE58009)/Homer(0.759)<br>More Information | Similar Motifs Found	motif file (matrix)
3	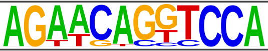	1e-15	-3.479e+01	0.17%	0.01%	132.5bp (125.2bp)	AR-halfsite(NR)/LNCaP-AR-ChIP-Seq(GSE27824)/Homer(0.576)<br>More Information | Similar Motifs Found	motif file (matrix)
4	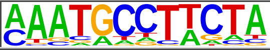	1e-14	-3.439e+01	0.12%	0.00%	133.7bp (0.0bp)	ZNF528(Zf)/HEK293-ZNF528.GFP-ChIP-Seq(GSE58341)/Homer(0.699)<br>More Information | Similar Motifs Found	motif file (matrix)
5	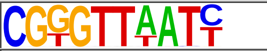	1e-14	-3.291e+01	0.28%	0.04%	118.2bp (99.3bp)	ZNF652/HepG2-ZNF652.Flag-ChIP-Seq(Encode)/Homer(0.701)<br>More Information | Similar Motifs Found	motif file (matrix)
6	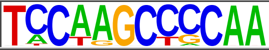	1e-13	-3.032e+01	0.21%	0.02%	96.6bp (122.8bp)	RUNX1(Runt)/Jurkat-RUNX1-ChIP-Seq(GSE29180)/Homer(0.562)<br>More Information | Similar Motifs Found	motif file (matrix)
7	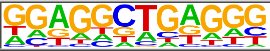	1e-12	-2.846e+01	5.71%	4.13%	115.9bp (101.2bp)	ETS:RUNX(ETS,Runt)/Jurkat-RUNX1-ChIP-Seq(GSE17954)/Homer(0.647)<br>More Information | Similar Motifs Found	motif file (matrix)
8	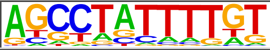	1e-12	-2.837e+01	0.44%	0.11%	123.1bp (89.3bp)	Mef2a(MADS)/HL1-Mef2a.biotin-ChIP-Seq(GSE21529)/Homer(0.641)<br>More Information | Similar Motifs Found	motif file (matrix)
9 *	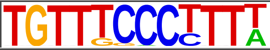	1e-11	-2.749e+01	0.40%	0.09%	133.0bp (92.8bp)	IRF3(IRF)/BMDM-Irf3-ChIP-Seq(GSE67343)/Homer(0.679)<br>More Information | Similar Motifs Found	motif file (matrix)
10 *	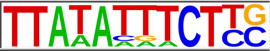	1e-11	-2.697e+01	0.12%	0.01%	126.6bp (72.4bp)	Six2(Homeobox)/NephronProgenitor-Six2-ChIP-Seq(GSE39837)/Homer(0.596)<br>More Information | Similar Motifs Found	motif file (matrix)
11 *	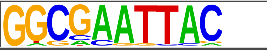	1e-11	-2.568e+01	0.43%	0.11%	118.1bp (98.2bp)	Lhx2(Homeobox)/HFSC-Lhx2-ChIP-Seq(GSE48068)/Homer(0.690)<br>More Information | Similar Motifs Found	motif file (matrix)
12 *	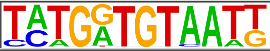	1e-11	-2.560e+01	0.19%	0.02%	109.0bp (100.7bp)	Chop(bZIP)/MEF-Chop-ChIP-Seq(GSE35681)/Homer(0.800)<br>More Information | Similar Motifs Found	motif file (matrix)
13 *	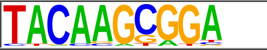	1e-10	-2.348e+01	0.56%	0.20%	97.4bp (97.4bp)	HINFP(Zf)/K562-HINFP.eGFP-ChIP-Seq(Encode)/Homer(0.560)<br>More Information | Similar Motifs Found	motif file (matrix)
14 *	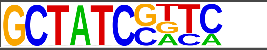	1e-9	-2.293e+01	0.09%	0.00%	152.9bp (63.8bp)	HNF6(Homeobox)/Liver-Hnf6-ChIP-Seq(ERP000394)/Homer(0.620)<br>More Information | Similar Motifs Found	motif file (matrix)
15 *	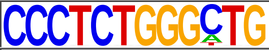	1e-9	-2.237e+01	0.14%	0.01%	135.6bp (75.1bp)	AP-2alpha(AP2)/Hela-AP2alpha-ChIP-Seq(GSE31477)/Homer(0.631)<br>More Information | Similar Motifs Found	motif file (matrix)
16 *	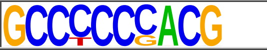	1e-9	-2.226e+01	0.33%	0.08%	105.3bp (92.8bp)	Egr2(Zf)/Thymocytes-Egr2-ChIP-Seq(GSE34254)/Homer(0.753)<br>More Information | Similar Motifs Found	motif file (matrix)
17 *	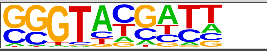	1e-9	-2.164e+01	0.18%	0.02%	155.9bp (111.5bp)	HINFP(Zf)/K562-HINFP.eGFP-ChIP-Seq(Encode)/Homer(0.588)<br>More Information | Similar Motifs Found	motif file (matrix)
18 *	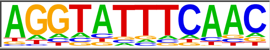	1e-9	-2.101e+01	0.39%	0.11%	106.5bp (104.9bp)	CHR(?)/Hela-CellCycle-Expression/Homer(0.560)<br>More Information | Similar Motifs Found	motif file (matrix)
19 *	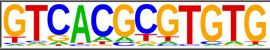	1e-8	-2.020e+01	0.14%	0.02%	73.9bp (53.5bp)	MNT(bHLH)/HepG2-MNT-ChIP-Seq(Encode)/Homer(0.629)<br>More Information | Similar Motifs Found	motif file (matrix)
20 *	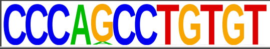	1e-8	-1.988e+01	0.11%	0.01%	80.1bp (102.2bp)	ZNF416(Zf)/HEK293-ZNF416.GFP-ChIP-Seq(GSE58341)/Homer(0.592)<br>More Information | Similar Motifs Found	motif file (matrix)
21 *	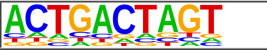	1e-8	-1.950e+01	29.01%	26.29%	122.5bp (100.3bp)	Bcl11a(Zf)/HSPC-BCL11A-ChIP-Seq(GSE104676)/Homer(0.553)<br>More Information | Similar Motifs Found	motif file (matrix)
22 *	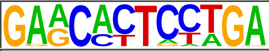	1e-7	-1.816e+01	0.17%	0.03%	99.8bp (87.1bp)	HRE(HSF)/Striatum-HSF1-ChIP-Seq(GSE38000)/Homer(0.693)<br>More Information | Similar Motifs Found	motif file (matrix)
23 *	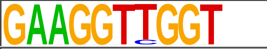	1e-7	-1.790e+01	0.13%	0.02%	148.1bp (40.7bp)	AMYB(HTH)/Testes-AMYB-ChIP-Seq(GSE44588)/Homer(0.555)<br>More Information | Similar Motifs Found	motif file (matrix)
24 *	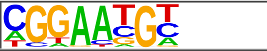	1e-7	-1.776e+01	11.41%	9.65%	121.7bp (99.3bp)	TEAD1(TEAD)/HepG2-TEAD1-ChIP-Seq(Encode)/Homer(0.858)<br>More Information | Similar Motifs Found	motif file (matrix)
25 *	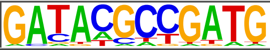	1e-7	-1.758e+01	0.09%	0.01%	116.0bp (24.1bp)	Gata6(Zf)/HUG1N-GATA6-ChIP-Seq(GSE51936)/Homer(0.555)<br>More Information | Similar Motifs Found	motif file (matrix)
26 *	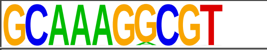	1e-6	-1.517e+01	0.11%	0.01%	107.0bp (121.2bp)	Sox3(HMG)/NPC-Sox3-ChIP-Seq(GSE33059)/Homer(0.599)<br>More Information | Similar Motifs Found	motif file (matrix)
27 *	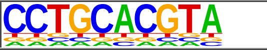	1e-6	-1.467e+01	0.08%	0.01%	127.7bp (50.0bp)	HIF-1a(bHLH)/MCF7-HIF1a-ChIP-Seq(GSE28352)/Homer(0.787)<br>More Information | Similar Motifs Found	motif file (matrix)
28 *	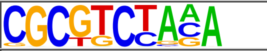	1e-6	-1.467e+01	0.08%	0.01%	72.7bp (55.9bp)	Smad4(MAD)/ESC-SMAD4-ChIP-Seq(GSE29422)/Homer(0.542)<br>More Information | Similar Motifs Found	motif file (matrix)
29 *	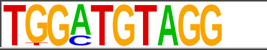	1e-6	-1.459e+01	0.33%	0.11%	105.9bp (100.1bp)	ETS:RUNX(ETS,Runt)/Jurkat-RUNX1-ChIP-Seq(GSE17954)/Homer(0.601)<br>More Information | Similar Motifs Found	motif file (matrix)
30 *	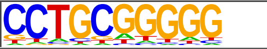	1e-6	-1.404e+01	15.28%	13.52%	122.8bp (102.2bp)	Egr1(Zf)/K562-Egr1-ChIP-Seq(GSE32465)/Homer(0.716)<br>More Information | Similar Motifs Found	motif file (matrix)
31 *	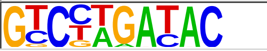	1e-6	-1.399e+01	0.21%	0.05%	105.0bp (104.4bp)	IRF4(IRF)/GM12878-IRF4-ChIP-Seq(GSE32465)/Homer(0.576)<br>More Information | Similar Motifs Found	motif file (matrix)

32 *	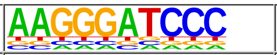	1e-5	-1.310e+01	0.17%	0.04%	106.9bp (113.9bp)	NFkB-p50,p52(RHD)/Monocyte-p50-ChIP-Chip(Schreiber_et_al.)/Homer(0.584)<br>More Information | Similar Motifs Found	motif file (matrix)
33 *	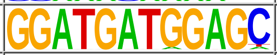	1e-5	-1.295e+01	0.10%	0.01%	248.0bp (88.4bp)	ZNF415(Zf)/HEK293-ZNF415.GFP-ChIP-Seq(GSE58341)/Homer(0.822)<br>More Information | Similar Motifs Found	motif file (matrix)
34 *	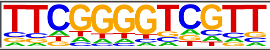	1e-5	-1.255e+01	0.06%	0.00%	77.3bp (0.0bp)	LRF(Zf)/Erythroblasts-ZBTB7A-ChIP-Seq(GSE74977)/Homer(0.691)<br>More Information | Similar Motifs Found	motif file (matrix)
35 *	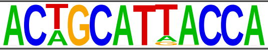	1e-5	-1.190e+01	0.07%	0.01%	118.9bp (42.5bp)	Hoxa10(Homeobox)/ChickenMSG-Hoxa10.Flag-ChIP-Seq(GSE86088)/Homer(0.586)<br>More Information | Similar Motifs Found	motif file (matrix)
36 *	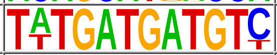	1e-5	-1.190e+01	0.07%	0.01%	195.1bp (84.6bp)	c-Jun-CRE(bZIP)/K562-cJun-ChIP-Seq(GSE31477)/Homer(0.682)<br>More Information | Similar Motifs Found	motif file (matrix)
37 *	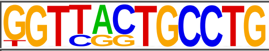	1e-5	-1.182e+01	0.12%	0.02%	103.8bp (78.1bp)	ERG(ETS)/VCaP-ERG-ChIP-Seq(GSE14097)/Homer(0.633)<br>More Information | Similar Motifs Found	motif file (matrix)
38 *	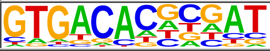	1e-2	-6.505e+00	0.03%	0.00%	87.3bp (0.0bp)	Tgif2(Homeobox)/mES-Tgif2-ChIP-Seq(GSE55404)/Homer(0.730)<br>More Information | Similar Motifs Found	motif file (matrix)
39 *	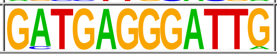	1e-2	-5.371e+00	0.04%	0.01%	96.1bp (88.1bp)	HOXA2(Homeobox)/mES-Hoxa2-ChIP-Seq(Donaldson_et_al.)/Homer(0.654)<br>More Information | Similar Motifs Found	motif file (matrix)

Homer *de novo* Motif Results (motif/20211105_h33_gwVsWt_noRep7_pnasParam_q10RmdupBiop_pj001_f0_up_osteo202103_inK4me1MinOl50/)

Known Motif Enrichment Results
Gene Ontology Enrichment Results
If Homer is having trouble matching a motif to a known motif, try copy/pasting the matrix file into STAMP
More information on motif finding results: HOMER | Description of Results | Tips
Total target sequences = 5240
Total background sequences = 44822
* - possible false positive

Rank	Motif	P-value	log P-pvalue	% of Targets	% of Background	STD(Bg STD)	Best Match/Details	Motif File
1	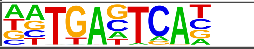	1e-42	-9.770e+01	10.42%	5.57%	135.5bp (102.7bp)	Fra1(bZIP)/BT549-Fra1-ChIP-Seq(GSE46166)/Homer(0.985)<br>More Information | Similar Motifs Found	motif file (matrix)
2	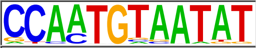	1e-23	-5.385e+01	0.50%	0.03%	103.8bp (106.7bp)	HOXB13/MA0901.2/Jaspar(0.610)<br>More Information | Similar Motifs Found	motif file (matrix)
3	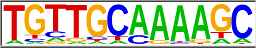	1e-19	-4.570e+01	3.24%	1.47%	113.0bp (102.1bp)	CEBP:AP1(bZIP)/ThioMac-CEBPb-ChIP-Seq(GSE21512)/Homer(0.764)<br>More Information | Similar Motifs Found	motif file (matrix)
4	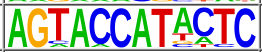	1e-19	-4.395e+01	0.29%	0.01%	112.6bp (96.9bp)	PB0137.1_Irf3_2/Jaspar(0.615)<br>More Information | Similar Motifs Found	motif file (matrix)
5	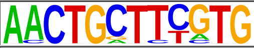	1e-17	-4.034e+01	0.38%	0.02%	111.4bp (97.9bp)	ETS:E-box(ETS,bHLH)/HPC7-Scl-ChIP-Seq(GSE22178)/Homer(0.647)<br>More Information | Similar Motifs Found	motif file (matrix)
6	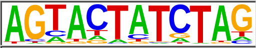	1e-16	-3.826e+01	1.93%	0.73%	135.3bp (100.9bp)	HRE(HSF)/Striatum-HSF1-ChIP-Seq(GSE38000)/Homer(0.595)<br>More Information | Similar Motifs Found	motif file (matrix)
7	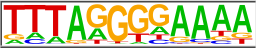	1e-16	-3.779e+01	8.76%	5.86%	128.5bp (102.5bp)	MZF1(var.2)/MA0057.1/Jaspar(0.677)<br>More Information | Similar Motifs Found	motif file (matrix)
8	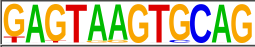	1e-16	-3.765e+01	0.23%	0.01%	133.3bp (24.3bp)	ISL2/MA0914.1/Jaspar(0.684)<br>More Information | Similar Motifs Found	motif file (matrix)
9	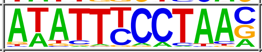	1e-15	-3.677e+01	0.94%	0.22%	104.4bp (103.2bp)	NFIL3/MA0025.2/Jaspar(0.693)<br>More Information | Similar Motifs Found	motif file (matrix)
10	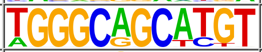	1e-15	-3.580e+01	0.36%	0.03%	123.7bp (114.9bp)	THAP1/MA0597.1/Jaspar(0.653)<br>More Information | Similar Motifs Found	motif file (matrix)
11	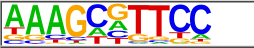	1e-15	-3.521e+01	16.28%	12.47%	126.9bp (104.3bp)	TEAD(TEA)/Fibroblast-PU.1-ChIP-Seq(Unpublished)/Homer(0.640)<br>More Information | Similar Motifs Found	motif file (matrix)
12	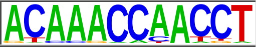	1e-14	-3.414e+01	0.42%	0.04%	105.4bp (107.8bp)	Sox9(HMG)/Limb-SOX9-ChIP-Seq(GSE73225)/Homer(0.608)<br>More Information | Similar Motifs Found	motif file (matrix)
13	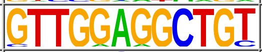	1e-14	-3.371e+01	0.21%	0.01%	108.8bp (93.4bp)	ZNF341/MA1655.1/Jaspar(0.605)<br>More Information | Similar Motifs Found	motif file (matrix)
14	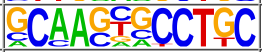	1e-14	-3.326e+01	0.27%	0.01%	105.2bp (88.3bp)	TCF12(var.2)/MA1648.1/Jaspar(0.627)<br>More Information | Similar Motifs Found	motif file (matrix)
15	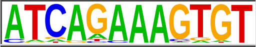	1e-13	-3.184e+01	0.61%	0.11%	126.3bp (102.9bp)	EHF/MA0598.3/Jaspar(0.682)<br>More Information | Similar Motifs Found	motif file (matrix)
16	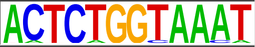	1e-13	-3.168e+01	0.29%	0.02%	101.0bp (94.9bp)	PH0048.1_Hoxa13/Jaspar(0.645)<br>More Information | Similar Motifs Found	motif file (matrix)
17	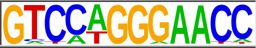	1e-13	-3.153e+01	0.48%	0.06%	99.3bp (98.2bp)	RBPJ/MA1116.1/Jaspar(0.671)<br>More Information | Similar Motifs Found	motif file (matrix)
18	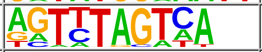	1e-12	-2.955e+01	1.95%	0.86%	110.5bp (101.9bp)	SMAD3/MA0795.1/Jaspar(0.720)<br>More Information | Similar Motifs Found	motif file (matrix)
19	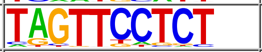	1e-12	-2.828e+01	3.91%	2.29%	121.1bp (104.5bp)	PB0058.1_Sfpi1_1/Jaspar(0.765)<br>More Information | Similar Motifs Found	motif file (matrix)
20	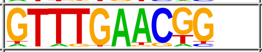	1e-12	-2.826e+01	0.92%	0.26%	150.9bp (106.7bp)	LIN54/MA0619.1/Jaspar(0.716)<br>More Information | Similar Motifs Found	motif file (matrix)
21	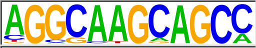	1e-12	-2.805e+01	0.46%	0.07%	129.5bp (112.9bp)	SD0002.1_at_AC_acceptor/Jaspar(0.691)<br>More Information | Similar Motifs Found	motif file (matrix)
22	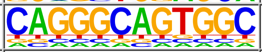	1e-12	-2.788e+01	0.15%	0.00%	79.2bp (0.0bp)	PB0091.1_Zbtb3_1/Jaspar(0.674)<br>More Information | Similar Motifs Found	motif file (matrix)
23	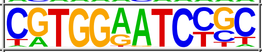	1e-12	-2.782e+01	0.25%	0.01%	80.1bp (105.4bp)	TEAD4(TEA)/Tropoblast-Tead4-ChIP-Seq(GSE37350)/Homer(0.719)<br>More Information | Similar Motifs Found	motif file (matrix)
24 *	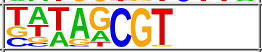	1e-11	-2.638e+01	3.89%	2.33%	133.8bp (104.7bp)	Ahr::Arnt/MA0006.1/Jaspar(0.680)<br>More Information | Similar Motifs Found	motif file (matrix)
25 *	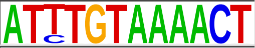	1e-11	-2.629e+01	0.21%	0.01%	86.2bp (47.0bp)	PB0187.1_Tcf7_2/Jaspar(0.639)<br>More Information | Similar Motifs Found	motif file (matrix)
26 *	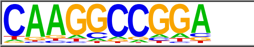	1e-11	-2.597e+01	6.95%	4.81%	127.8bp (101.4bp)	NR5A1/MA1540.1/Jaspar(0.726)<br>More Information | Similar Motifs Found	motif file (matrix)
27 *	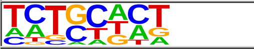	1e-11	-2.576e+01	19.89%	16.33%	127.2bp (102.8bp)	NFIL3/MA0025.2/Jaspar(0.602)<br>More Information | Similar Motifs Found	motif file (matrix)
28 *	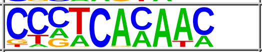	1e-11	-2.553e+01	2.65%	1.42%	111.7bp (99.9bp)	PB0120.1_Foxj1_2/Jaspar(0.684)<br>More Information | Similar Motifs Found	motif file (matrix)
29 *	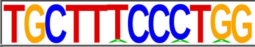	1e-10	-2.489e+01	0.23%	0.01%	114.2bp (109.0bp)	ZNF528/MA1597.1/Jaspar(0.668)<br>More Information | Similar Motifs Found	motif file (matrix)
30 *	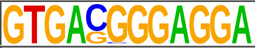	1e-10	-2.429e+01	0.25%	0.02%	107.6bp (91.4bp)	ZNF263/MA0528.2/Jaspar(0.766)<br>More Information | Similar Motifs Found	motif file (matrix)
31 *	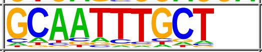	1e-9	-2.256e+01	8.15%	5.98%	125.2bp (104.3bp)	PH0024.1_Dlx5/Jaspar(0.646)<br>More Information | Similar Motifs Found	motif file (matrix)
32 *	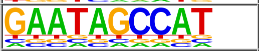	1e-9	-2.176e+01	0.27%	0.03%	123.5bp (81.3bp)	ZNF341(Zf)/EBV-ZNF341-ChIP-Seq(GSE113194)/Homer(0.713)<br>More Information | Similar Motifs Found	motif file (matrix)
33 *	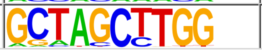	1e-9	-2.106e+01	0.74%	0.23%	121.1bp (101.7bp)	Nr2e3/MA0164.1/Jaspar(0.664)<br>More Information | Similar Motifs Found	motif file (matrix)

34 *	TATCCTTCTA	1e-8	-2.019e+01	0.50%	0.12%	104.8bp (101.4bp)	SD0003.1_at_AC_acceptor/Jaspar(0.722)<br>More Information | Similar Motifs Found	motif file (matrix)
35 *	ACCCGTTAAT	1e-8	-1.956e+01	0.11%	0.00%	47.9bp (139.2bp)	PB0046.1_Mybl1_1/Jaspar(0.744)<br>More Information | Similar Motifs Found	motif file (matrix)
36 *	GGGTACTAGA	1e-8	-1.890e+01	0.13%	0.01%	73.8bp (86.0bp)	ZBTB12(Zf)/HEK293-ZBTB12.GFP-ChIP-Seq(GSE58341)/Homer(0.753)<br>More Information | Similar Motifs Found	motif file (matrix)
37 *	AGCCGCTGCTCC	1e-8	-1.890e+01	0.13%	0.01%	65.8bp (22.6bp)	Ascl2/MA0816.1/Jaspar(0.705)<br>More Information | Similar Motifs Found	motif file (matrix)
38 *	AGGTTGAAAT	1e-7	-1.803e+01	0.32%	0.06%	129.2bp (97.6bp)	CHR(?)/Hela-CellCycle-Expression/Homer(0.666)<br>More Information | Similar Motifs Found	motif file (matrix)
39 *	ACTCACACGC	1e-6	-1.555e+01	1.70%	0.94%	111.3bp (98.5bp)	Ahr::Arnt/MA0006.1/Jaspar(0.739)<br>More Information | Similar Motifs Found	motif file (matrix)
40 *	GCAACTAGGC	1e-6	-1.550e+01	0.11%	0.00%	85.5bp (58.2bp)	PB0159.1_Rfx4_2/Jaspar(0.688)<br>More Information | Similar Motifs Found	motif file (matrix)
41 *	TACGAAAGGA	1e-6	-1.443e+01	4.28%	3.04%	120.0bp (102.6bp)	PB0106.1_Arid5a_2/Jaspar(0.639)<br>More Information | Similar Motifs Found	motif file (matrix)
42 *	CAGAGCTCTGCT	1e-5	-1.186e+01	0.08%	0.00%	49.5bp (75.3bp)	PB0099.1_Zfp691_1/Jaspar(0.595)<br>More Information | Similar Motifs Found	motif file (matrix)
43 *	CTAGTATT	1e-5	-1.184e+01	3.70%	2.68%	129.1bp (102.0bp)	PB0106.1_Arid5a_2/Jaspar(0.715)<br>More Information | Similar Motifs Found	motif file (matrix)
44 *	CTTTGATT	1e-4	-1.034e+01	2.56%	1.78%	107.5bp (104.1bp)	TCF7/MA0769.2/Jaspar(0.876)<br>More Information | Similar Motifs Found	motif file (matrix)

Homer *de novo* Motif Results (motif/20211105_h33_gwVsWt_noRep7_pnasParam_q10RmdupBiop_pj001_f0_up_osteo202103_inGenhMinOl50/)

Known Motif Enrichment Results
Gene Ontology Enrichment Results
If Homer is having trouble matching a motif to a known motif, try copy/pasting the matrix file into STAMP
More information on motif finding results: HOMER | Description of Results | Tips
Total target sequences = 397
Total background sequences = 49499
* - possible false positive

Rank	Motif	P-value	log P-pvalue	% of Targets	% of Background	STD(Bg STD)	Best Match/Details	Motif File
1	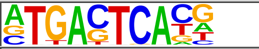	1e-18	-4.371e+01	22.92%	8.08%	142.4bp (112.4bp)	JunB(bZIP)/DendriticCells-Junb-ChIP-Seq(GSE36099)/Homer(0.973)<br>More Information | Similar Motifs Found	motif file (matrix)
2	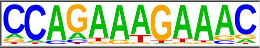	1e-13	-3.201e+01	8.56%	1.65%	107.7bp (109.1bp)	bZIP:IRF(bZIP,IRF)/Th17-BatF-ChIP-Seq(GSE39756)/Homer(0.696)<br>More Information | Similar Motifs Found	motif file (matrix)
3	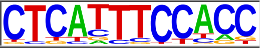	1e-13	-3.062e+01	7.56%	1.33%	110.0bp (112.0bp)	ZKSCAN5/MA1652.1/Jaspar(0.758)<br>More Information | Similar Motifs Found	motif file (matrix)
4	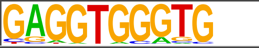	1e-12	-2.886e+01	11.84%	3.43%	133.6bp (111.8bp)	KLF9/MA1107.2/Jaspar(0.720)<br>More Information | Similar Motifs Found	motif file (matrix)
5 *	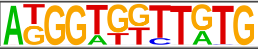	1e-10	-2.442e+01	3.27%	0.24%	130.0bp (112.1bp)	PB0120.1_Foxj1_2/Jaspar(0.634)<br>More Information | Similar Motifs Found	motif file (matrix)
6 *	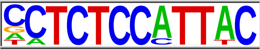	1e-10	-2.402e+01	2.02%	0.05%	114.9bp (82.0bp)	Isl1/MA1608.1/Jaspar(0.660)<br>More Information | Similar Motifs Found	motif file (matrix)
7 *	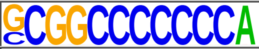	1e-9	-2.250e+01	1.01%	0.00%	84.9bp (0.0bp)	ZNF740/MA0753.2/Jaspar(0.788)<br>More Information | Similar Motifs Found	motif file (matrix)
8 *	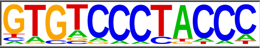	1e-9	-2.250e+01	1.01%	0.00%	142.5bp (0.0bp)	KLF4/MA0039.4/Jaspar(0.708)<br>More Information | Similar Motifs Found	motif file (matrix)
9 *	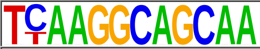	1e-9	-2.204e+01	1.26%	0.01%	72.8bp (44.4bp)	PB0041.1_Mafb_1/Jaspar(0.660)<br>More Information | Similar Motifs Found	motif file (matrix)
10 *	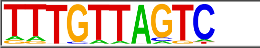	1e-9	-2.166e+01	4.53%	0.67%	100.3bp (111.0bp)	PB0119.1_Foxa2_2/Jaspar(0.809)<br>More Information | Similar Motifs Found	motif file (matrix)
11 *	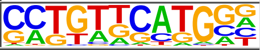	1e-9	-2.127e+01	4.03%	0.52%	121.9bp (112.3bp)	AR-halfsite(NR)/LNCaP-AR-ChIP-Seq(GSE27824)/Homer(0.686)<br>More Information | Similar Motifs Found	motif file (matrix)
12 *	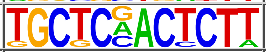	1e-8	-2.057e+01	1.76%	0.05%	60.8bp (110.5bp)	ZNF652/MA1657.1/Jaspar(0.616)<br>More Information | Similar Motifs Found	motif file (matrix)
13 *	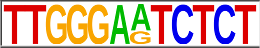	1e-8	-1.974e+01	1.01%	0.00%	179.8bp (93.0bp)	RBPJ/MA1116.1/Jaspar(0.628)<br>More Information | Similar Motifs Found	motif file (matrix)
14 *	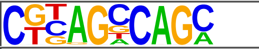	1e-8	-1.943e+01	4.03%	0.59%	118.3bp (106.8bp)	MafA(bZIP)/Islet-MafA-ChIP-Seq(GSE30298)/Homer(0.688)<br>More Information | Similar Motifs Found	motif file (matrix)
15 *	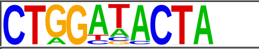	1e-8	-1.935e+01	2.77%	0.23%	103.4bp (111.7bp)	PB0159.1_Rfx4_2/Jaspar(0.636)<br>More Information | Similar Motifs Found	motif file (matrix)
16 *	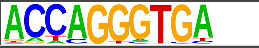	1e-8	-1.895e+01	6.80%	1.79%	146.8bp (112.6bp)	PB0200.1_Zfp187_2/Jaspar(0.607)<br>More Information | Similar Motifs Found	motif file (matrix)
17 *	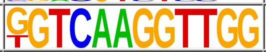	1e-7	-1.812e+01	1.01%	0.01%	24.2bp (88.4bp)	RAR:RXR(NR),DR0/ES-RAR-ChIP-Seq(GSE56893)/Homer(0.713)<br>More Information | Similar Motifs Found	motif file (matrix)
18 *	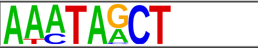	1e-7	-1.799e+01	17.63%	8.74%	127.1bp (111.1bp)	Mef2c(MADS)/GM12878-Mef2c-ChIP-Seq(GSE32465)/Homer(0.757)<br>More Information | Similar Motifs Found	motif file (matrix)
19 *	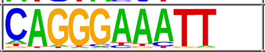	1e-7	-1.784e+01	8.31%	2.69%	130.4bp (110.0bp)	RELB/MA1117.1/Jaspar(0.706)<br>More Information | Similar Motifs Found	motif file (matrix)
20 *	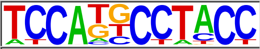	1e-7	-1.749e+01	3.53%	0.51%	89.9bp (106.4bp)	ZFX(Zf)/mES-Zfx-ChIP-Seq(GSE11431)/Homer(0.630)<br>More Information | Similar Motifs Found	motif file (matrix)
21 *	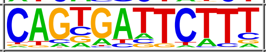	1e-7	-1.737e+01	11.84%	4.88%	121.0bp (111.3bp)	Gfi1b(Zf)/HPC7-Gfi1b-ChIP-Seq(GSE22178)/Homer(0.668)<br>More Information | Similar Motifs Found	motif file (matrix)
22 *	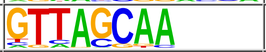	1e-7	-1.647e+01	17.13%	8.72%	129.4bp (110.9bp)	PB0041.1_Mafb_1/Jaspar(0.735)<br>More Information | Similar Motifs Found	motif file (matrix)
23 *	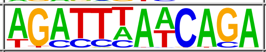	1e-6	-1.592e+01	2.77%	0.33%	106.6bp (108.6bp)	PB0016.1_Foxj1_1/Jaspar(0.598)<br>More Information | Similar Motifs Found	motif file (matrix)
24 *	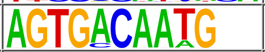	1e-6	-1.505e+01	2.52%	0.28%	52.6bp (115.7bp)	Sox17/MA0078.1/Jaspar(0.763)<br>More Information | Similar Motifs Found	motif file (matrix)
25 *	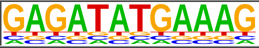	1e-6	-1.421e+01	0.76%	0.01%	106.5bp (77.2bp)	T1ISRE(IRF)/ThioMac-Ifnb-Expression/Homer(0.713)<br>More Information | Similar Motifs Found	motif file (matrix)
26 *	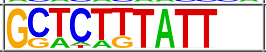	1e-5	-1.222e+01	2.52%	0.39%	133.4bp (114.6bp)	CDX2/MA0465.2/Jaspar(0.704)<br>More Information | Similar Motifs Found	motif file (matrix)
27 *	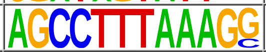	1e-5	-1.214e+01	0.76%	0.01%	38.2bp (127.5bp)	TATA-Box(TBP)/Promoter/Homer(0.679)<br>More Information | Similar Motifs Found	motif file (matrix)
28 *	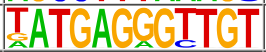	1e-4	-1.151e+01	1.01%	0.03%	52.5bp (104.8bp)	PB0024.1_Gcm1_1/Jaspar(0.678)<br>More Information | Similar Motifs Found	motif file (matrix)
29 *	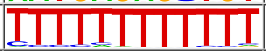	1e-4	-1.067e+01	6.55%	2.61%	123.4bp (117.5bp)	PB0182.1_Srf_2/Jaspar(0.841)<br>More Information | Similar Motifs Found	motif file (matrix)
30 *	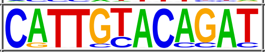	1e-2	-4.830e+00	0.25%	0.00%	25.5bp (0.0bp)	SOX2/MA0143.4/Jaspar(0.662)<br>More Information | Similar Motifs Found	motif file (matrix)
31 *	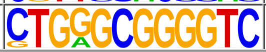	1e-2	-4.830e+00	0.25%	0.00%	53.6bp (0.0bp)	PB0039.1_Klf7_1/Jaspar(0.786)<br>More Information | Similar Motifs Found	motif file (matrix)

Homer *de novo* Motif Results (motif/20211105_h33_gwVsWt_noRep7_pnasParam_q10RmdupBiop_pj001_f0_up_osteo202103_inPromtorDiyMinOl50/)

Known Motif Enrichment Results
Gene Ontology Enrichment Results
If Homer is having trouble matching a motif to a known motif, try copy/pasting the matrix file into STAMP
More information on motif finding results: HOMER | Description of Results | Tips
Total target sequences = 2633
Total background sequences = 47292
* - possible false positive

Rank	Motif	P-value	log P-pvalue	% of Targets	% of Background	STD(Bg STD)	Best Match/Details	Motif File
1	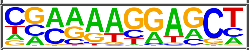	1e-53	-1.232e+02	1.10%	0.01%	46.4bp (125.9bp)	POL013.1_MED-1/Jaspar(0.592)<br>More Information | Similar Motifs Found	motif file (matrix)
2	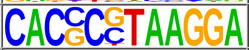	1e-51	-1.184e+02	0.99%	0.01%	90.3bp (97.1bp)	PB0180.1_Sp4_2/Jaspar(0.596)<br>More Information | Similar Motifs Found	motif file (matrix)
3	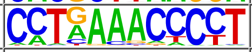	1e-49	-1.138e+02	2.32%	0.15%	143.4bp (113.8bp)	IRF4(IRF)/GM12878-IRF4-ChIP-Seq(GSE32465)/Homer(0.630)<br>More Information | Similar Motifs Found	motif file (matrix)
4	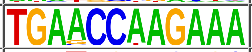	1e-49	-1.130e+02	1.03%	0.01%	44.0bp (139.6bp)	ZNF136/MA1588.1/Jaspar(0.701)<br>More Information | Similar Motifs Found	motif file (matrix)
5	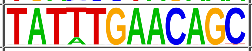	1e-46	-1.079e+02	0.99%	0.01%	132.4bp (73.6bp)	ZNF341/MA1655.1/Jaspar(0.727)<br>More Information | Similar Motifs Found	motif file (matrix)
6	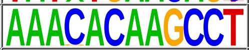	1e-46	-1.060e+02	0.80%	0.00%	106.1bp (48.1bp)	OSR2/MA1646.1/Jaspar(0.650)<br>More Information | Similar Motifs Found	motif file (matrix)
7	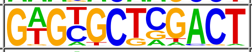	1e-46	-1.060e+02	0.80%	0.00%	18.0bp (0.0bp)	TCFL5/MA0632.2/Jaspar(0.593)<br>More Information | Similar Motifs Found	motif file (matrix)
8	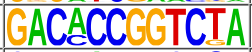	1e-43	-1.001e+02	0.76%	0.00%	31.3bp (0.0bp)	PKNOX2/MA0783.1/Jaspar(0.669)<br>More Information | Similar Motifs Found	motif file (matrix)
9	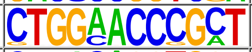	1e-42	-9.782e+01	0.91%	0.01%	91.8bp (73.9bp)	ZBTB12/MA1649.1/Jaspar(0.757)<br>More Information | Similar Motifs Found	motif file (matrix)
10	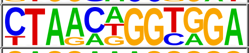	1e-39	-9.164e+01	1.60%	0.08%	102.7bp (79.7bp)	SNAI3/MA1559.1/Jaspar(0.662)<br>More Information | Similar Motifs Found	motif file (matrix)
11	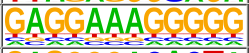	1e-37	-8.600e+01	1.10%	0.03%	90.9bp (93.5bp)	Etv2(ETS)/ES-ER71-ChIP-Seq(GSE59402)/Homer(0.594)<br>More Information | Similar Motifs Found	motif file (matrix)
12	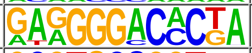	1e-37	-8.566e+01	0.91%	0.01%	58.2bp (98.8bp)	PLAGL2/MA1548.1/Jaspar(0.660)<br>More Information | Similar Motifs Found	motif file (matrix)
13	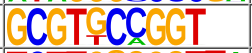	1e-35	-8.107e+01	0.72%	0.01%	82.5bp (51.4bp)	Ahr::Arnt/MA0006.1/Jaspar(0.679)<br>More Information | Similar Motifs Found	motif file (matrix)
14	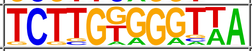	1e-33	-7.686e+01	0.61%	0.00%	75.5bp (0.0bp)	PB0201.1_Zfp281_2/Jaspar(0.642)<br>More Information | Similar Motifs Found	motif file (matrix)
15	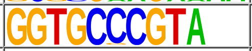	1e-32	-7.593e+01	0.68%	0.01%	70.7bp (62.9bp)	THAP1/MA0597.1/Jaspar(0.709)<br>More Information | Similar Motifs Found	motif file (matrix)
16	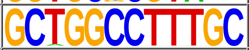	1e-32	-7.593e+01	0.68%	0.01%	94.5bp (50.2bp)	Erra(NR)/HepG2-Erra-ChIP-Seq(GSE31477)/Homer(0.768)<br>More Information | Similar Motifs Found	motif file (matrix)
17	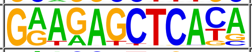	1e-32	-7.454e+01	0.91%	0.02%	13.6bp (114.3bp)	NR1I3/MA1534.1/Jaspar(0.667)<br>More Information | Similar Motifs Found	motif file (matrix)
18	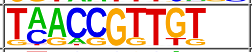	1e-32	-7.431e+01	1.18%	0.05%	92.8bp (104.3bp)	PB0046.1_Mybl1_1/Jaspar(0.744)<br>More Information | Similar Motifs Found	motif file (matrix)
19	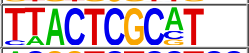	1e-31	-7.292e+01	1.18%	0.05%	116.3bp (108.1bp)	GCM2/MA0767.1/Jaspar(0.706)<br>More Information | Similar Motifs Found	motif file (matrix)
20	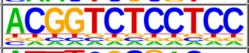	1e-31	-7.250e+01	0.76%	0.01%	40.5bp (135.5bp)	ZNF263/MA0528.2/Jaspar(0.654)<br>More Information | Similar Motifs Found	motif file (matrix)
21	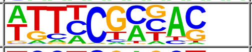	1e-31	-7.242e+01	0.80%	0.01%	76.8bp (67.9bp)	CEBPB/MA0466.2/Jaspar(0.659)<br>More Information | Similar Motifs Found	motif file (matrix)
22	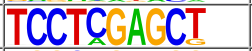	1e-31	-7.242e+01	1.48%	0.10%	106.9bp (107.2bp)	ZBTB6/MA1581.1/Jaspar(0.722)<br>More Information | Similar Motifs Found	motif file (matrix)
23	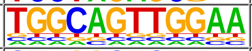	1e-30	-7.120e+01	0.57%	0.00%	120.4bp (0.0bp)	AMYB(HTH)/Testes-AMYB-ChIP-Seq(GSE44588)/Homer(0.831)<br>More Information | Similar Motifs Found	motif file (matrix)
24	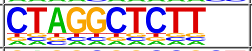	1e-30	-7.103e+01	1.14%	0.05%	130.6bp (95.2bp)	ZNF341(Zf)/EBV-ZNF341-ChIP-Seq(GSE113194)/Homer(0.641)<br>More Information | Similar Motifs Found	motif file (matrix)
25	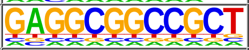	1e-30	-7.085e+01	0.65%	0.01%	23.5bp (70.7bp)	HINFP/MA0131.2/Jaspar(0.608)<br>More Information | Similar Motifs Found	motif file (matrix)
26	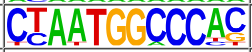	1e-30	-6.949e+01	0.84%	0.02%	56.6bp (117.5bp)	Isl1/MA1608.1/Jaspar(0.755)<br>More Information | Similar Motifs Found	motif file (matrix)
27	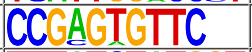	1e-30	-6.914e+01	1.10%	0.04%	169.0bp (107.1bp)	PB0139.1_Irf5_2/Jaspar(0.634)<br>More Information | Similar Motifs Found	motif file (matrix)
28	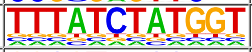	1e-28	-6.561e+01	0.53%	0.00%	0.0bp (0.0bp)	TRPS1(Zf)/MCF7-TRPS1-ChIP-Seq(GSE107013)/Homer(0.675)<br>More Information | Similar Motifs Found	motif file (matrix)
29	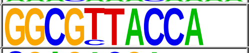	1e-27	-6.316e+01	0.91%	0.03%	28.8bp (104.6bp)	PAX5/MA0014.3/Jaspar(0.703)<br>More Information | Similar Motifs Found	motif file (matrix)
30	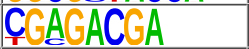	1e-26	-6.180e+01	2.62%	0.50%	87.9bp (110.0bp)	Smad2(MAD)/ES-SMAD2-ChIP-Seq(GSE29422)/Homer(0.650)<br>More Information | Similar Motifs Found	motif file (matrix)
31	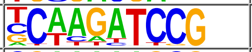	1e-26	-6.030e+01	1.60%	0.17%	136.8bp (96.5bp)	SPDEF/MA0686.1/Jaspar(0.674)<br>More Information | Similar Motifs Found	motif file (matrix)
32	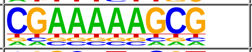	1e-26	-6.008e+01	0.49%	0.00%	0.3bp (0.0bp)	PRDM1/MA0508.3/Jaspar(0.623)<br>More Information | Similar Motifs Found	motif file (matrix)
33	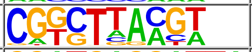	1e-24	-5.626e+01	1.41%	0.14%	146.1bp (108.3bp)	OTX1/MA0711.1/Jaspar(0.610)<br>More Information | Similar Motifs Found	motif file (matrix)
34	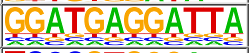	1e-24	-5.595e+01	0.53%	0.01%	0.0bp (43.0bp)	Crx/MA0467.1/Jaspar(0.734)<br>More Information | Similar Motifs Found	motif file (matrix)
35	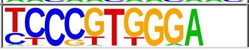	1e-24	-5.564e+01	4.52%	1.51%	96.4bp (106.8bp)	Ebf2/MA1604.1/Jaspar(0.856)<br>More Information | Similar Motifs Found	motif file (matrix)

36	CGCCATTGTA	1e-24	-5.543e+01	0.65%	0.01%	40.6bp (102.5bp)	SOX2/MA0143.4/Jaspar(0.787)<br>More Information | Similar Motifs Found	motif file (matrix)
37	ATACTACTCC	1e-23	-5.463e+01	0.46%	0.00%	0.0bp (36.3bp)	OTX2/MA0712.2/Jaspar(0.619)<br>More Information | Similar Motifs Found	motif file (matrix)
38	ATCCTTTCGT	1e-23	-5.365e+01	0.68%	0.02%	127.3bp (123.1bp)	SD0003.1_at_AC_acceptor/Jaspar(0.621)<br>More Information | Similar Motifs Found	motif file (matrix)
39	CGGGTAGG	1e-21	-5.012e+01	20.71%	13.78%	151.5bp (110.3bp)	ZNF263/MA0528.2/Jaspar(0.681)<br>More Information | Similar Motifs Found	motif file (matrix)
40	CCCGAAGG	1e-21	-4.978e+01	11.59%	6.47%	135.3bp (115.8bp)	TFAP2A(var.2)/MA0810.1/Jaspar(0.689)<br>More Information | Similar Motifs Found	motif file (matrix)
41	TCGGTCTT	1e-20	-4.743e+01	12.92%	7.60%	114.3bp (106.7bp)	NR1H4/MA1110.1/Jaspar(0.630)<br>More Information | Similar Motifs Found	motif file (matrix)
42	CCCATTCG	1e-19	-4.543e+01	2.70%	0.72%	109.2bp (120.6bp)	NFY(CCAAT)/Promoter/Homer(0.680)<br>More Information | Similar Motifs Found	motif file (matrix)
43	TGCGTGCA	1e-19	-4.413e+01	6.80%	3.23%	113.2bp (105.6bp)	Ahr::Arnt/MA0006.1/Jaspar(0.823)<br>More Information | Similar Motifs Found	motif file (matrix)
44	TATGGGTT	1e-17	-4.008e+01	12.54%	7.69%	114.9bp (104.4bp)	MF0009.1_TRP(MYB)_class/Jaspar(0.784)<br>More Information | Similar Motifs Found	motif file (matrix)
45	GCATCATC	1e-17	-3.944e+01	4.86%	2.08%	131.0bp (102.6bp)	ATF4/MA0833.2/Jaspar(0.748)<br>More Information | Similar Motifs Found	motif file (matrix)
46 *	GCCCCCCA	1e-11	-2.725e+01	6.50%	3.66%	115.3bp (116.2bp)	VEZF1/MA1578.1/Jaspar(0.852)<br>More Information | Similar Motifs Found	motif file (matrix)
47 *	ACAAGCTG	1e-11	-2.703e+01	4.98%	2.56%	127.1bp (100.5bp)	Nr2e3/MA0164.1/Jaspar(0.695)<br>More Information | Similar Motifs Found	motif file (matrix)
48 *	TTCTCCCC	1e-10	-2.342e+01	7.37%	4.53%	121.9bp (108.2bp)	ZNF263/MA0528.2/Jaspar(0.783)<br>More Information | Similar Motifs Found	motif file (matrix)
49 *	AAAGGTAG	1e-9	-2.251e+01	3.27%	1.53%	140.3bp (109.3bp)	ETV4/MA0764.2/Jaspar(0.677)<br>More Information | Similar Motifs Found	motif file (matrix)
50 *	GTTTTGTC	1e-5	-1.255e+01	2.05%	1.04%	115.3bp (109.5bp)	Sox21(HMG)/ESC-SOX21-ChIP-Seq(GSE110505)/Homer(0.694)<br>More Information | Similar Motifs Found	motif file (matrix)
