# Supplementary material for: Aberrant paracrine signalling for bone remodelling underlies the mutant histone-driven giant cell tumour of bone
Source: Cell Death Differ. 2022 Aug 3;29(12):2459–71. doi: 10.1038/s41418-022-01031-x (PMC9750984; doi:10.1038/s41418-022-01031-x)
Supplement: Supplementary file 12 — Author contribution form [file 41418_2022_1031_MOESM12_ESM.pdf]

# DECLARATION OF CONTRIBUTIONS TO ARTICLE

**ADMC**

| Manuscript Number: | Journal Name: | |
|---|---|---|
| CDD-21-1129R | *Cell Death & Differentiation* | (the 'Journal') |

Proposed Title of the Contribution:

Aberrant paracrine signalling for bone remodelling underlies the mutant histone-driven giant cell tumour of bone (the 'Contribution)

Author(s):

Lucia Cottone, Lorena Ligammari, Helen J. Knowles, Hang-Mao Lee, Stephen Henderson, Sara Bianco, Christopher Davies, Sandra Strauss, Fernanda Amary, Ana Paula Leite, Roberto Tirabosco, Kristian Haendler, Joachim L. Schultze, Javier Herrero, Paul O'Donnell, Agamemnon E. Grigoriadis, Paolo Salomoni and Adrienne M. Flanagan (the 'Authors')

For all *CDD* articles, each person named as an author in the published version must be able to show he or she has contributed substantially to the article.

Authorship credit should be based on 1) substantial contributions to conception and design, acquisition of data, or analysis and interpretation of data; 2) drafting the article or revising it critically for important intellectual content; and 3) final approval of the version to be published. Authors should meet conditions 1, 2 and 3.

Any person who cannot be shown to have made a substantial contribution to the article cannot be listed as an author in the final version. The name of any person who is deemed to have made a minor contribution can, however, appear in the Acknowledgments section of the article.

Please complete the table below to indicate the contributions of all named authors to the manuscript.

| Author Full Name: | Specification of Contribution to the Manuscript: |
|---|---|
| Lucia Cottone | Acquisition of data, analysis and interpretation of data, substantial contributions to conception and design, drafting the article or revising it critically for important intellectual content |
| Lorena Ligammari, Helen J. Knowles | Acquisition of data, analysis and interpretation of data |
| Sara Bianco, Christopher Davies | Acquisition of data |
| Ana Paula Leite, Hang-Mao Lee, Stephen Henderson, Javier Herrero | Bioinformatic analysis |
| Kristian Haendler,Joachim L. Schultze | Acquisition of data |
| Sandra Strauss, Fernanda Amary, Roberto Tirabosco, Paul O'Donnell | Clinical data and sample curation |
| Agamemnon E. Grigoriadis | Drafting the article or revising it critically for important intellectual content |
| Paolo Salomoni and Adrienne M. Flanagan | Substantial contributions to conception and design, Drafting the article or revising it critically for important intellectual conten, final approval of the version to be published |
| | |
| | |
| | |
| | |
| | |

Please complete the table below to indicate the contributions of all named authors to the figures.

Figure 1:

Acquisition of data, analysis and interpretation of data: Lucia Cottone, Lorena Ligammari, Helen J. Knowles, Sara Bianco, Christopher Davies,
Clinical data and sample curation: Sandra Strauss, Fernanda Amary, Roberto Tirabosco, Paul O'Donnell
Substantial contributions to conception and design: Paolo Salomoni and Adrienne M. Flanagan
Drafting the article or revising it critically for important intellectual content: Agamemnon E. Grigoriadis

Figure 2:

Acquisition of data, analysis and interpretation of data: Lucia Cottone, Lorena Ligammari, Sara Bianco, Christopher Davies
Bioinformatic analysis: Ana Paula Leite, Stephen Henderson, Javier Herrero
Clinical data and sample curation: Sandra Strauss, Fernanda Amary, Roberto Tirabosco
Substantial contributions to conception and design: Paolo Salomoni and Adrienne M. Flanagan

Figure 3:

Acquisition of data, analysis and interpretation of data: Lucia Cottone, Lorena Ligammari, Kristian Haendler,Joachim L. Schultze
Bioinformatic analysis: Hang-Mao Lee
Substantial contributions to conception and design: Paolo Salomoni and Adrienne M. Flanagan

Figure 4:

Acquisition of data, analysis and interpretation of data: Lucia Cottone, Lorena Ligammari, Kristian Haendler,Joachim L. Schultze
Bioinformatic analysis: Hang-Mao Lee
Substantial contributions to conception and design: Paolo Salomoni and Adrienne M. Flanagan

Figure 5:

Acquisition of data, analysis and interpretation of data: Lucia Cottone, Lorena Ligammari, Helen J. Knowles
Substantial contributions to conception and design: Paolo Salomoni and Adrienne M. Flanagan

Figure 6:

Acquisition of data, analysis and interpretation of data: Lucia Cottone, Lorena Ligammari, Helen J. Knowles, Christopher Davies.
Clinical data and sample curation: Sandra Strauss, Paul O'Donnell
Substantial contributions to conception and design: Paolo Salomoni and Adrienne M. Flanagan
Drafting the article or revising it critically for important intellectual content: Agamemnon E. Grigoriadis

| Signed for and on behalf of the Author(s): | Print Name: | Date: |
| --- | --- | --- |
| Lucia Cottone | Lucia Cottone | 14/12/2021 |
